# Marker-assisted trait enhancement for drought tolerance and bacterial leaf blight resistance in rice cultivar HUR-917

**DOI:** 10.3389/fpls.2026.1848543

**Published:** 2026-07-14

**Authors:** Pandurang B. Arsode, Prakash Singh, Shravan Kumar Singh, Ravi Pratap Singh, Manish Kumar, Debarchana Jena, Diptibala Rout, Tanayashree Devalaxmi, Somyashree Mishra, Vineeta Singh, Namrata, Jawahar Lal Katara, Sanghamitra Samantaray, Ramlakhan Verma, Vijai Pal Bhadana

**Affiliations:** 1 Department of Genetics and Plant Breeding, Institute of Agricultural Sciences, Banaras Hindu University, Varanasi, Uttar Pradesh, India; 2 Crop Improvement Division, ICAR-Central Rice Research Institute, Cuttack, Odisha, India; 3 Department of Genetics and Plant Breeding, BAU-Botanical Research Unit, Bikramganj, Rohtas (Bihar Agricultural University), Bihar, India; 4 Department of Genetics and Plant Breeding, Rajasthan Agricultural Research Institute (Sri Karan Narendra Agriculture University, Jobner), Durgapura, Rajasthan, India; 5 Dr. B R Chaudhary Agricultural Research Station, Agriculture University, Jodhpur, Rajasthan, India; 6 ICAR-Indian Institute of Agricultual Biotechnology, Ranchi, Jharkhand, India

**Keywords:** bacterial blight resistance, drought tolerance, HUR-917, marker assisted backcross breeding, SSR markers

## Abstract

Moisture stress and bacterial blight (BB) are major constraints limiting rice productivity in irrigated and rainfed shallow lowland ecosystems. Despite the identification of several drought-yield quantitative trait loci (QTLs) and BB resistance genes, their combined deployment in aromatic short-grain rice cultivars remains limited. The popular aromatic cultivar HUR-917, a restorer line HUR-917, a restorer line to reproductive-stage drought and BB, highlighting the need for developing climate-resilient and disease-resistant versions without compromising yield and grain quality. The present study aimed to improve HUR-917 through marker-assisted introgression of drought-yield QTLs *qDTY2.2* and *qDTY4*.1 along with BB resistance genes *xa5, xa13*, and *Xa21*. DRR Dhan-42 was used as the donor for drought QTLs, while improved HUR-917 (HUR-917-15-2-2-1) served as the donor for BB resistance genes, with HUR-917 used as the recurrent parent (RP). Marker-assisted backcross breeding (MABB) was employed for precise introgression of target loci and accelerated recovery of the RP genome (RPG) using gene-linked markers and 98 polymorphic SSR markers. A total of 16 BC_3_F_4_ near-isogenic lines (NILs) carrying the target QTLs and BB resistance genes were developed, with more than 90% RPG while retaining the product profile of HUR-917. Among them, NILs HR-12-1-4-87-5-2-4, HR-12-1-4-87-5-2-8, HR-12-1-4-87-5-2-32, HR-12-1-4-87-295-4-19, HR-12-70-9-258-169-1-8, and HR-12-70-9-258-223-5–12 exhibited stable performance under both drought stress and irrigated conditions. Under moisture stress, the NILs showed comparatively lower reductions in plant height, spikelet fertility, and grain yield than the RP, primarily due to improved spikelet fertility and higher grain number per panicle. Importantly, no yield penalty was observed under non-stress conditions. The improved NILs also retained desirable grain quality attributes, including aroma, amylose content (AC), and head rice recovery (HRR). Physiological analyses further confirmed enhanced drought tolerance, as the NILs maintained higher chlorophyll content and accumulated substantially greater proline levels (average, 77.06 mg g^-^¹) compared with the RP. Overall, the combined introgression of *qDTY2*.2, *qDTY4*.1, *xa5, xa13*, and *Xa21* successfully enhanced drought tolerance, BB resistance, and yield stability without adversely affecting grain quality. The developed multi-stress-tolerant NILs constitute valuable genetic resources for breeding climate-resilient aromatic rice cultivars suited to the Indo-Gangetic Plains.

## 1 Introduction

Rice (*Oryza sativa* L.) is a staple food for over half of the global population, particularly in Asia (Das et al., 2022; Katara et al., 2020). It supplies a significant portion of dietary carbohydrates for nearly half of the world’s population and contributing over 20% of their daily caloric intake (Maclean et al., 2002; Kumar et al., 2023). In India, rice accounts for more than 40% of the total food grain production (Verma et al., 2017). As a semi-aquatic plant, rice has traditionally relied on abundant water for optimal growth and development. However, frequent occurrences of drought amid changing climate and irregular rainfall patterns are challenging traditional cultivation practices. Enhancing drought tolerance in rice is therefore crucial for ensuring stable food supplies and agricultural sustainability. By 2050, the global population is projected to reach 9.0 billion, with approximately 17.8% of this population living in the Indian subcontinent (Godfray et al., 2010). Ensuring food security for this growing population will be increasingly difficult due to the depletion of resources like arable land and irrigation water. Additionally, climate change, with its unpredictable and unseasonal weather patterns leading to crop failures due to resurgence of biotic stresses, further complicates the challenge of feeding a rapidly expanding population. Consequently, there is a continuous need to prioritize rice improvement efforts focused on enhancing yield and sustainability under biotic and abiotic stresses.

Drought, an osmotic stress, is one of the major abiotic constraints affecting rice productivity worldwide. Bacterial leaf blight (BLB) is among the most devastating biotic diseases in rice, causing severe reductions in grain yield. India experienced 22 severe drought years from 1981 to 2012 primarily as a consequence of a 50%–75% reduction in average rainfall per annum during its prime southwest monsoon season (Gautam and Bana, 2014). Rice is susceptible to drought stress at all growth stages; however, moisture stress during the reproductive stage is particularly critical, as it has the most severe impact on grain yield (Barnabás et al., 2008). Vegetative and intermittent drought spells occur during the early wet season, especially in an area receiving bimodal rainfall, while intermittent drought is caused by irregular rainfall events. Drought stress adversely affects rice growth by reducing plant height (PH), biomass accumulation, and the translocation of metabolites from source tissues to grains (Kumar et al., 2006).

Under terminal drought, maintaining high tissue water potential is essential for the growth and development of reproductive organs. Ensuring an adequate grain sink size and conserving water are considered important mechanisms for drought tolerance (Fukai et al., 1999). The reproductive development in plant is highly vulnerable to limiting moisture (Saini and Westgate, 1999). Yambao and Ingram (1988) reported that drought stress for 15 days at the panicle initiation stage reduced rice yield by up to 70%. Yield reductions of 88% and 52% were observed when drought occurred during the flowering and grain filling stages, respectively. GY is generally considered to be the most important parameter for rice farmers in rainfed lowland areas where drought developed late in the wet season (Cooper, 1999).

Developing drought-tolerant cultivars would involve changing the aquatic nature of rice into that of an aerobic one (Price and Courtois, 1999). However, drought is a complex trait as timing, duration, and intensity of drought are unpredictable. Earlier efforts to improve rice yield under drought, which mainly focused on improving secondary traits such as root morphology, leaf water potential, panicle water potential, osmotic homeostasis, and relative water content, have made limited success (Fukai et al., 1999). Thus, the selection and incorporation of yield quantitative trait loci (QTLs) under drought (*qDTY*) is gaining paramount importance for the trait of interest like yield, which is less heritable and can be introgressed by using marker-assisted backcross breeding (MABB). Because of the complexity of drought stress and the range of conditions to which rainfed rice crops are exposed, a multidimensional breeding approach including marker-assisted selection is worthy to improve drought tolerance in rice (Vikram et al., 2015).

A large-scale systematic study using several mapping populations identified major QTLs associated with grain yield under drought stress (Kumar et al., 2018). These QTLs have been introgressed into rice cultivars to develop drought-tolerant varieties. Some of the identified QTLs have been reported to be effective across ecosystems, whereas few were specific to a particular ecosystem. Out of the several QTLs reported for reproductive stage drought tolerance in rice, *qDTY1.1* (Vikram et al., 2011; Ghimire et al., 2012), *qDTY2.*1, *qDTY3.1* (Dixit et al., 2014; Venuprasad et al., 2009), *qDTY2.2* (Swamy et al., 2013), *qDTY 4.1* (Swamy et al., 2013), and *qDTY12.1* (Bernier et al., 2009) showed consistent grain yield under drought across different genetic backgrounds and have been used in breeding applications. Major drought-yield QTLs, including *qDTY1.1, qDTY2.1, qDTY2.2, qDTY3.1, qDTY3.2, qDTY4.1, qDTY6.1, qDTY10.1*, and *qDTY12*.1, have been successfully introgressed into several elite rice cultivars through marker-assisted breeding approaches. These include CR Dhan 801 (*qDTY1.1, qDTY2.1*, and *qDTY3.1*), CR Dhan 802 (*qDTY2.1* and *qDTY3.1*), CR Dhan 804 (*qDTY2.2*), CR Dhan 808 (*qDTY12.1* and *qDTY3.1*), Pusa 44 (*qDTY2.1, qDTY3.1*, and *qDTY12.1*), MTU 1010 (*qDTY2.2* and *qDTY4.1*), and NLR34449 (*qDTY2.2* and *qDTY4.1*), leading to the development of rice varieties with enhanced reproductive-stage (RS) drought tolerance (Mishra and Pandey, 2013; Sandhu et al., 2019; Muthu et al., 2020; Bhukya et al., 2020; Dwivedi et al., 2021; Dhawan et al., 2021). Bacterial blight (BB) is a major constraint to rice production worldwide, affecting almost all rice ecosystems and regions and causing yield losses ranging from 20% to complete crop failure (Chen et al., 1997; Chu et al., 2006; Das et al., 2022; Kumar et al., 2023). The disease occurs as leaf blight and the systemic wilt phase known as Kresek are favored by warm temperatures (25–30°C), high humidity, heavy rainfall, water stagnation, excessive nitrogen fertilization, dense planting, and wind-induced leaf injuries (Chukwu et al., 2019; Cobb et al., 2019). The causal pathogen, *Xanthomonas oryzae* pv. *oryzae* (*Xoo*), enters through wounds and colonizes xylem tissues. *Xoo* populations are highly diverse and dynamic, producing multiple virulence factors including extracellular polysaccharides, hydrolytic enzymes, siderophores, and type III secretion system-dependent effectors that suppress host immunity (Eamchit and Mew, 1982; Doyle and Doyle, 1987; DRR, 2011; Ellur et al., 2016; Das et al., 2018; Dash et al., 2016). To date, approximately 30 *Xoo* races have been reported globally (Doyle and Doyle, 1987; Eram et al., 2014; Fahy and Persley, 1983).

Host resistance achieved through the deployment of resistance (R) genes remains the most effective and sustainable strategy for BB management (FAOSTAT, 2018; Kumar et al., 2023; Das et al., 2022). Advances in genomics and molecular breeding have enabled precise mapping and pyramiding of R genes into elite rice cultivars (Das et al., 2018; Ghosh et al., 1971; Gnanamanickam et al., 1999; Hsu et al., 2020; Iyer and McCouch, 2004; Kumar et al., 2023; Das et al., 2022). More than 40 BB resistance genes have been identified, with *Xa4, xa5, Xa7, xa13*, *Xa21*, and *Xa38* being the most widely utilized (Ghosh et al., 1971; Gnanamanickam et al., 1999; Hsu et al., 2020; Iyer and McCouch, 2004). Functional gene combinations, such as *xa*5 with *Xa7* or *Xa21* and *xa13* with *Xa21*, have demonstrated enhanced and broad-spectrum resistance, particularly against virulent Indian *Xoo* races (Ghosh et al., 1971; Jones and Dangl, 2006; Joseph et al., 2004; Juliano, 1971). Among these, Xa21, encoding NBS-LRR (Nucleotide binding site–Leucine-rich repeats) protein, has been extensively deployed owing to its durable and broad resistance (Ghosh et al., 1971; Gnanamanickam et al., 1999; Hsu et al., 2020; Kauffman et al., 1973; Khan et al., 2003).

Pyramiding functionally complementary R genes provides greater durability and resistance breadth than single-gene deployment (Ghosh et al., 1971; Gnanamanickam et al., 1999; Hsu et al., 2020; Khanna et al., 2015). In eastern India, a BB hotspot, gene combinations including *Xa21 + xa13 + xa5* (+ *Xa4*) has proven highly effective against diverse *Xoo* populations (Ghosh et al., 1971; Gnanamanickam et al., 1999; Hsu et al., 2020). The integration of advanced molecular breeding tools such as marker-assisted selection, marker-assisted backcrossing, and genomic selection has further accelerated the development of BB-resistant rice cultivars with high precision and efficiency (Ghosh et al., 1971; Kim et al., 2015).

In view of the above considerations and based on success of previous QTL-gene pyramiding studies, the current breeding efforts were made to combine drought and BB resistance in elite aromatic rice cultivar HUR-917. Stepwise crossing has been used to combine traits from two different donors, and the MABB approach was applied to track alleles at different generations.

## 2 Materials and methods

### 2.1 Experimental material and breeding approach

**Figure 1:**
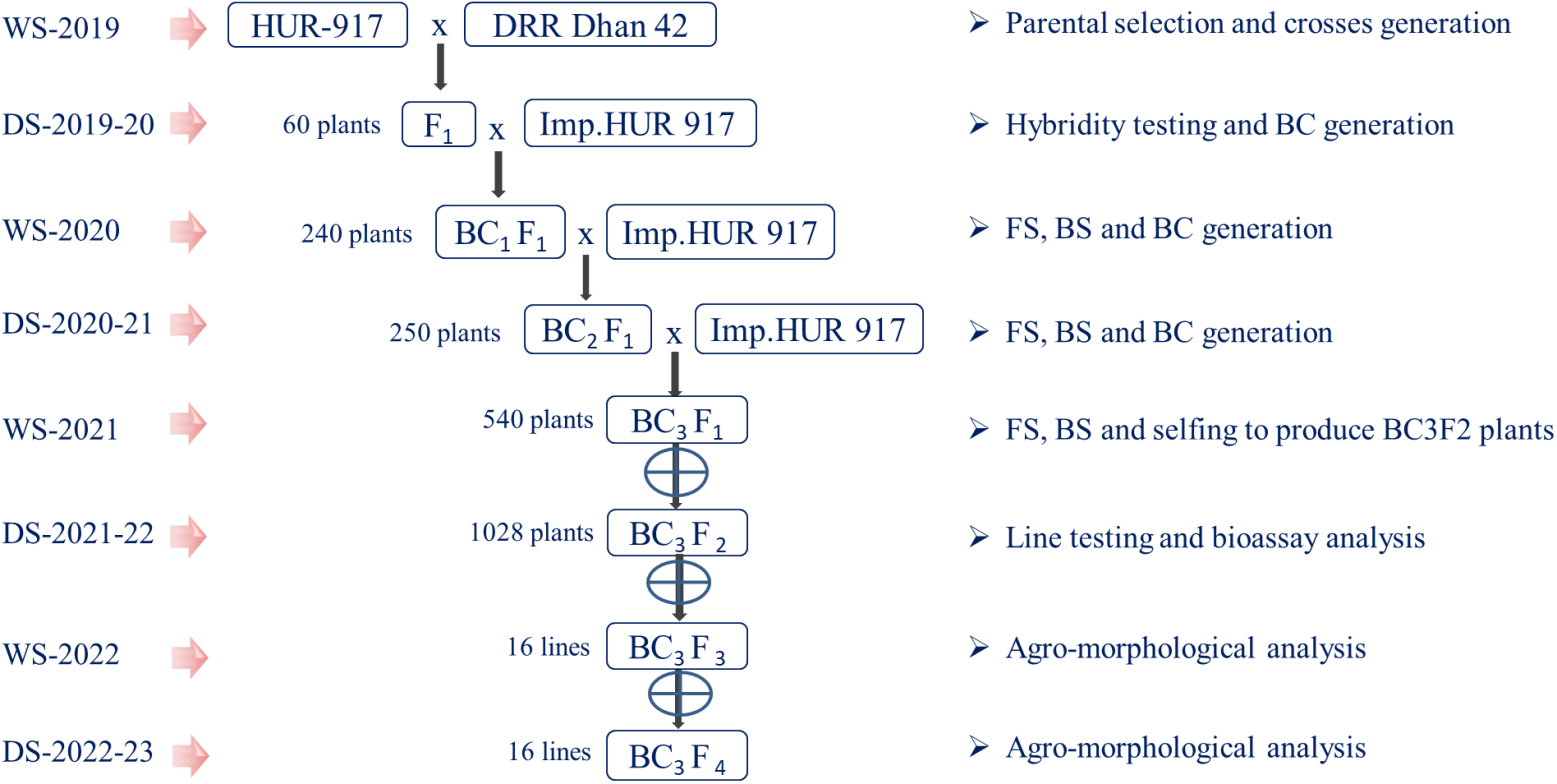
Marker-aided breeding strategy for trait improvement in HUR-917. FS, foreground selection; BS, background selection; BC, backcrossing.

The drought-tolerant variety DRR Dhan-42, carrying the yield QTLs (*qDTY2.2* and *qDTY4.1*), and improved HUR-917-15-2-2-1 (*Xa21, xa13*, and *xa5*) (Kumar et al, 2023), taken as donor (have genetic similarity i.e. 0.77 with RP) parents for marker-assisted improvement of reproductive stage drought tolerance (RSDT) and BB resistance in the widely cultivated rice variety HUR-917 (**Table 1**; **Figure 1**; **Supplementary Table 1**; **Supplementary Figure 1**). HUR-917 is a semi-dwarf, highly aromatic short grain, medium slender, high-yielding, medium maturity (135–140 days) duration variety. It has an intermediate (23.75%) amylose content (AC) with excellent cooking quality, which is comparable to the grain quality characteristics of Lalmati, Kalanamak, Badshahbhog, and Dubraj premium rice varieties. HUR-917 has a milling value of 68.8% and a head rice recovery (HRR) of 65.3%. It is semi-dwarf with stiffy stems and has a strong tillering ability. It is cultivated in the irrigated Indo-Gangetic plain zone of India especially Uttar Pradesh and the adjoining states of U.P. such as a Bihar, M.P., and Chhattisgarh (Singh R. P. et al., 2015). HUR-917 is preferred by farmers for its superior grain quality, aroma, and high irrigated yield, but its susceptibility to drought and BB requires genetic improvement for climate resilience.

**Table 1:** Parental description of *indica* rice cultivar, its pedigree and features.

Genotype	Pedigree	Special features	Yield (t/ha)	Duration (days)	Recommendation for cultivation
HUR-917	Dehradun Basmati Selection-13	High yielding, semi dwarf (105–115 cm), resistance to lodging and neck blast disease and good grain quality. Susceptible to BB and drought	4.2–4.5	130–135	Notified for cultivation in Eastern India during 2015
DRR Dhan-42	Aday sel x IR-64	NIL of IR64, pyramided with 2 QTLs (*qDTY 2.2* and *qDTY 4.1*) having high yield under drought stress at both vegetative and reproductive stages.	2-2.5 tons/ha under drought conditions and 4.5 to 5 tons/ha under irrigated conditions	120–125	Notified for cultivation in IR 64 growing areas
Improved HUR-917 (HUR-917-15-2-2-1)	HUR-917/IRBB-66 (HUR-917-15-2-2-1)	NIL-HUR-917 having BB resistance pyramided with *xa5, xa13*, and *Xa21*		130–135	Improved HUR-917 NIL (Kumar et al., 2023)

Hybridity of individual F_1_ plants of cross HUR-917 × DRR Dhan-42 was validated using tightly linked molecular markers RM279 and RM518 (Swamy et al., 2013; Sandhu and Kumar, 2017), along with pTA248 (Khush, 2005), xa13-prom (Singh et al., 2011; Das et al., 2022 and Kumar et al., 2023), and RM122 (Chen et al., 1997) (**Table 2**). Confirmed F_1_ hybrids were subsequently backcrossed with the recurrent parent (RP) to generate the BC_1_F_1_ population. The introgression of target genes along with THE recovery of the RP genome was monitored in each backcross generation using linked foreground markers and 98 polymorphic SSR markers for background analysis (**Supplementary Table 3**), combined with detailed phenotypic assessment. Marker data were further utilized to evaluate genetic relationships among parental lines and derived near-isogenic lines (NILs) through THE DARwin 6.0 software (Perrier and Jacquemoud-Collet, 2017). In the BC_1_F_1_ generation, plants showing maximum recovery of the RP genome together with desirable phenotypic characteristics, days to 50% flowering (DFF), PH, grain length-to-breadth (L/B) ratio, HRR, and AC were selected and advanced to subsequent generations. A total of 12 BC_3_F_1_ plants carrying heterozygous alleles for the targeted loci were self-pollinated to produce the BC_3_F_2_ generation. The BC_3_F_2_ population was further subjected to FS, BS, and phenotypic screening to identify individuals combining maximum recovery of the RP genome with the desired phenotypic profile. In each backcross and BC_3_F_2_ generation, selected plants were advanced using the single seed descent (SSD) approach under field-based rapid generation advance (RGA) conditions to expedite the development of NILs. Selection differential (Δ*d*) for important product profile traits, including DFF (days), PH (cm), grain L/B ratio, HRR, and AC content, was estimated across backcross and segregating generations. The developed 32 BC_3_F_3_ and 16 BC_3_F_4_ NILs, along with parents, were evaluated for drought tolerance using the Standard Evaluation System (SES) protocol (Singh et al., 2022; Venuprasad et al., 2009; Vikram et al., 2011; International Rice Research Institute (IRRI), 2013) at the research farm of ICAR-Central Rice Research Institute, India. Biochemical traits such as total chlorophyll content (Witham et al., 1971) and proline accumulation (Bates et al., 1973) were also measured to determine physiological responses associated with the introgressed drought QTLs under moisture stress conditions. In addition, resistance to BB was assessed through bioassays involving eight virulent isolates of *Xoo*. Disease severity was quantified by estimating the percent disease index (PDI) and area under the disease progress curve (AUDPC) to evaluate the extent of improvement achieved in the derived lines (Pradhan et al., 2019).

**Table 2:** Gene-specific/linked markers used for **forward** and recombinant selection.

Gene/QTL	Chrom. no.	Linked SSR marker	Physical location (Mb)	Type	Marker sequence	Reference
*qDTY_2.2_*	2	RM279	2.88	FS	CCTCTCACTCACGTGGACTCTCC CCTCACCCTAGGCTTTGATATGC	Swamy et al., 2013; Sandhu and Kumar, 2017
		RM236	2.10	RS	GTGAAGCACATGTGGCTAGTTGC TTCCCTCAAGAATCTGTGTCTTCC	
		RM555	4.30	RS	TTGACATGCGAAATGGAGATGG TTGGATCAGCCAAAGGAGACC	
*qDTY_4.1_*	4	RM518	2.02	FS	AAGACACAAGCAAACAGCTCAACC AAGCTTGCTTGGTTCAAGAGAGG	
		RM335	0.68	RS	GTACACACCCACATCGAGAAGC TCCATGGATATACGAGGAGATGC	
		RM16368	2.44	RS	TGTCCAGAGAATGACAAAGTACGC GGATGTATATCTGCCACCAAATGC	
*xa5*	5	RM122	1.65	FS	GCACTGCAACCATCAATGAATC CCTAGGAGAAACTAGCCGTCCA	Chen et al., 1997; Das et al., 2022
		RM17941	3.40	RS	GCCTCGAAGAACCAGTAGAACAGC CTTGTCTTCTCCTCCTCCTGTGC	
*xa13*	8	xa13prom	1.65	FS	GGCCATGGCTCAGTGTTTAT GAGCTCCAGCTCTCCAAATG	
		RM23356	24.20	RS	GCCTCCAACAGATCTCCTATCTGG TTTGGCGCTAATGAGAGATTGG	Singh et al., 2011; Dash et al., 2016; Das et al., 2022
		RM22914	30.90	RS	CCAATCATTAACCCCTGAGC GCCTTCATGCTTCAGAAGAC	
*Xa21*	11	pTA248	22.60	FS	AGACGCGGAAGGGTGGTTCCCGGA AGACGCGGTAATCGAAAGATGAAA	Khush, 2005; Das et al., 2022; Kumar et al., 2023
		RM26969	21.50	RS	CTCACACTTGCAACATCCTAGC AAGGCTCTAGTTGGTGAAGACC	
*BADH2*	8	FMbadh2-E7	82.80	FS	GGTTGCATTTACTGGGAGTT CAGTGAAACAGGCTGTCAAG	Shi et al., 2008; Dinkar et al., 2024

### 2.2 PCR and marker analysis

The genomic DNA from the leaf sample of 21-day-old seedlings was isolated and purified using the CTAB (cetyl trimethyl ammonium bromide) method (Doyle and Doyle, 1990). The polymerase chain reaction (PCR) was carried out using 15 ng of template DNA, 1× Taq assay-buffer, 0.3 mM MgCl_2_, 133.0 µM dNTPs, 1 U/µL Taq DNA polymerase (Thermo-scientific, Life Science Products, India), and 1.25 µM of each primer (Eurofins, Genomics, Hyderabad, India). The Eppendorf Thermo Cycler (USA) was used for PCR with the following program: (1) denaturation at 94 °C for 3 min; (2) 39 cycles for denaturation for 30 s at 94 °C, annealing for 30 s at 56 °C, and extension for 1 min at 72 °C; and (3) final extension at 72 °C for 5 min. The amplified products were resolved with 2.5% MetaphorTM Agarose gel (Typhoon FLA 700, Alpha Innotech, USA) and visualized under a UV light source in a photographed gel documentation (Gel-Doc) system (Gel DocTM XR, Bio-Rad Laboratories Inc., USA).

The presence of targeted loci in the F_1_ and subsequent backcross generations was monitored using tightly linked molecular markers. The SSR marker RM279, located approximately 2.8 cM from *qDTY2*.2, and RM518, positioned 2.02 cM from qDTY4.1 (Swamy et al., 2013; Sandhu and Kumar, 2017), were employed for tracking drought-tolerant QTLs. For BB resistance genes, pTA248 (0.2 cM from *Xa21*) (Khush, 2005; Pradhan et al., 2015) xa13-prom (co-segregating with *xa13*, 0 cM) (Kumar et al., 2023; Singh et al., 2011) and RM122 (0.4 cM from *xa5*) (Chen et al., 1997; Sundaram et al., 2008) were used for foreground selection (**Table 2**). Recovery of the RP genome was assessed using 98 polymorphic SSR markers distributed across the rice genome (**Table 3**; **Supplementary Table 3**, **Supplementary Table 5B**–M). These markers were selected from an initial set of 480 SSRs based on polymorphism detected between HUR-917 and DRR Dhan-42, ensuring uniform genome coverage. Recombinant selection around the target loci was performed using additional flanking markers, including RM236 and RM555 for *qDTY2*.2, and RM335 and RM16365 for *qDTY4*.1. Similarly, markers RM26969 (linked to *Xa21*), RM23356 and RM22914 (associated with *xa13*), and RM17941 (linked to *xa5*) were used to confirm recombination events around the BB resistance genes located on chromosomes 11, 8, and 5, while the drought QTLs were positioned on chromosomes 2 and 4. The marker data obtained in the BC_3_F_4_ generation were further utilized to construct graphical genotypes of individual NILs using the Graphical Genotyper (GGT 2.0) software (Van Berloo, 2008) to visualize the extent of donor and RP genome in the improved lines (2022-23).

**Table 3:** Summary of BC generations and RP genome recovery in NILs of HUR-917.

Generation	Total plants raised	Gene positive plants ¶	Plants advanced	% RPG	Selection criteria
F1	60	48	4	*	Hybridity testing gene linked markers
BC1F1	240	14	5	67.40–79.57	FS, BS, phenomics, and bioassay
BC2F1	250	18	8	78.57–87.56	BS, phenomics, and bioassay
BC3F1	540	12	2	82.65–91.25	BS, phenomics, and bioassay
BC3F2	1,028	16	3	90.39–93.0	BS, phenomics, and bioassay
BC3F3	16 families (each 120 plants)	16 families (32 plants)	16 NILs (16 plants)	>90	Phenomics and screening/bioassay analysis
BC3F4	16 NILs	16 NILs	16 NILs	>90	Phenomics and screening/bioassay analysis

*Not estimated; RPG, recurrent parent genome; ¶ number of plants/lines positive for qDTY2.2 + qDTY4.1 +Xa21+xa13+xa5; FS, foreground selection; BS, background selection.

### 2.3 Phenotypic evaluation of NILs under NS and RSDT conditions

Phenotypic evaluation of the NILs (BC_3_F_3_ and BC_3_F_4_) carrying *qDTY2.2+qDTY4.1+ Xa21+xa13+xa5* was carried out in two consecutive seasons under non-stress (NS) and RS drought environments following recommended agronomic practices at the research farm of ICAR-Central Rice Research Institute, India. A set of 32 BC_3_F_3_ NILs, along with the parental lines, was evaluated under irrigated and RSDT conditions using a randomized complete block design (RCBD) with three replications. The data of 16 BC3F3 NILs that had maximum RP genome and phenome were considered for analysis, and a set of selected 16 NILs was further evaluated in the BC3F4 generation. Standard crop management practices were followed throughout the experiment. Drought stress was imposed at the reproductive stage using a rainout shelter (ROS) facility, while 20 cm × 15 cm plant spacing was maintained in both irrigated and stress environments. The crop was irrigated regularly until the panicle initiation stage, after which irrigation was discontinued to create moisture stress. Water was drained from the field approximately 30 days after transplanting, and irrigation was withheld for 25–30 days to establish a drought cycle during the reproductive phase. Stress conditions were maintained until leaf rolling symptoms were observed in the RP, indicating severe water deficit. During this period, soil moisture tension (SMT) decreased to approximately −50 kPa at 30 cm soil depth and −70 kPa at 15 cm depth, corresponding to soil moisture levels of approximately 13% and 15%, respectively. Measurements of SMT and soil moisture content were recorded weekly to monitor the intensity of drought stress. The drought evaluation protocol followed established methodologies reported in previous studies (International Rice Research Institute (IRRI), 2013).

The data were recorded on five plants from each of the entries for the characters, namely, DFF, days to maturity (DM), PH, flag leaf length (FLL), flag leaf width (FLW), number of effective tillers per plant (NETPP), panicle length (PL), number of grains per panicle (NGP), test weight (TW), grain yield per plant (GYPP), drought susceptibility (DS), leaf rolling, proline content (mg g^−1^), and chlorophyll content. The NILs carrying all targeted genes/QTLs and resistant/tolerant to BLB and drought were selected for grain and cooking quality analysis for the parameters such as hulling, milling, HRR, chalkiness, kernel length before cooking (KLBC), kernel breadth before cooking (KBBC), L/B ratio, kernel length after cooking (KLAC), elongation ratio (ER), volume expansion ratio, alkali spreading value, AC (Juliano, 1971), GC content (Ghosh et al., 1971), and aroma. All procedures involving plant material adhered to relevant institutional, national, and international guidelines and legislation.

Experimental observations were analyzed using analysis of variance (ANOVA) under the General Linear Model (GLM) framework to evaluate the effects of treatments and their associated sources of variation. The statistical model adopted was:


$$Y_{ij}=\;\mu \;+\;T_{i}+\;B_{j}+\;\epsilon_{ij}$$

where *Y_ij_* denotes the measured value of the *i*th treatment in the *j*th replication, *μ* represents the grand mean, *T_i_* corresponds to the treatment effect, *B_j_* denotes the replication (block) effect, and *ϵ_ij_* is the residual error term, assumed to follow an independent normal distribution with a mean of zero and homogeneous variance. The significance of treatment effects was tested using the *F*-test at the 5% probability level (*p* ≤ 0.05). When significant differences were detected, treatment means were separated using the Least Significant Difference (LSD) test and Duncan’s Multiple Range Test (DMRT).

Statistical analyses were performed using PBTools v1.4 (International Rice Research Institute (IRRI), 2014), SPSS (IBM Corp., 2019), and XLSTAT (Addinsoft, 2023). Molecular analyses such as dendrogram construction, similarity matrix estimation, and graphical representation of trait introgression and background marker recovery were carried out using DARwin-6.0 (Perrier and Jacquemoud-Collet, 2017), R package version 1.0.7 (Kassambara and Mundt, 2020; R Core Team, 2023), and GGT 2.0 (Van Berloo, 2008).

### 2.4 BB bioassay analysis

NILs and successive backcross (BC) progenies carrying resistance (R) gene combinations in either heterozygous or homozygous states were cultivated under open field conditions together with their respective recurrent and donor parents. These materials were phenotypically evaluated against eight highly virulent *Xoo* races (Xa17, Xa7, xa2, Xb7, Xc4, xd1, xa1, and xa5) prevalent in the eastern region of the country. The pathogen isolates were maintained on peptone sucrose agar (PSA) medium (Fahy and Persley, 1983), and pathogenicity assays were conducted using single-colony-derived bacterial suspensions standardized to 10^8^ cfu mL^-^¹. For inoculation, the leaf-clipping method was employed by cutting the uppermost five leaves of each plant (Kauffman et al., 1973). Plants were examined at 24-h intervals to record the onset and progression of disease symptoms. Lesion length was measured at 14, 21, and 28 days after inoculation (DAI) (Mondal et al., 2014), and disease reactions were rated on a 0–9 scale (International Rice Research Institute (IRRI), 2010), with lesions ≥15 cm categorized as highly susceptible. Disease intensity and temporal dynamics were quantified by calculating epidemiological indices, including the PDI and the AUDPC (Gnanamanickam et al., 1999).

## 3 Results

### 3.1 Molecular profiling and donor selection

A total of 480 SSR markers were screened across HUR-917 (RP), DRR Dhan-42 (*qDTY2*.2 and *qDTY4*.1), Dhagaddeshi (*qDTY1.1*), Nagina-22 (*qDTY1.1, qDTY3.2*, and *qDTY10.1*), and Vandana (*qDTY6*.1). Among these, 98 markers (20.4%) exhibited polymorphism between the RP and the donors (**Supplementary Table 3**). Genetic diversity analysis indicated varying levels of relatedness among the parents, with similarity indices ranging from 0.17 to 0.77 (**Supplementary Table 1**; **Supplementary Figure 1**). DRR Dhan-42, carrying *qDTY2.2* and *qDTY4.1*, displayed the highest genetic similarity with HUR-917 (0.77), making it an ideal donor for trait introgression. Bioassay evaluation of the improved line HUR 917 (HUR-917-15-2-2-1), which carries Xa21, xa13, and xa5, and retains over 94% of the RP genome, exhibited resistant reactions against eight Xoo races, with lesion lengths ranging from 0.95 ± 0.68 cm to 3.44 ± 0.47 cm (**Table 4**). To minimize undesirable linkage drag (LD) and facilitate rapid fixation of segregating loci in progenies, DRR Dhan-42 [74] and HUR-917-15-2-2–1 [73] were selected as donors for drought tolerance and BB resistance genes in our trait improvement program.

**Table 4:** Disease reaction (lesion length after 21 days of inoculation) in improved NILs (BC_3_F_3_) carrying resistance genes against eight different races of *Xoo*.

Pyramided lines (NILs)	Gene combination	Disease lesion length (LL) in cm (mean ± standard error)									
		xa1	xa2	Xa7	xb7	xc4	xd1	xa5	Xa17	Mean LL	Disease reaction
HR-12-1-4-87-5-2-4	Xa21+xa13+xa5+ qDTY2.2+ qDTY24.1	3.35 ± 0.43	1.94 ± 0.24	3.52 ± 0.16	2.16 ± 0.37	3.28 ± 0.74	1.53 ± 0.44	2.57 ± 0.29	2.21 ± 0.45	2.46	R
HR-12-1-4-87-5-2-8	Xa21+xa13+xa5+ qDTY2.2+ qDTY24.1	3.72 ± 0.68	2.02 ± 0.78	3.28 ± 0.38	2.40 ± 0.91	3.48 ± 0.09	1.96 ± 0.21	2.61 ± 0.33	2.35 ± 0.49	2.73	R
HR-12-1-4-87-5-2-32	Xa21+xa13+xa5+ qDTY2.2+ qDTY24.1	3.74 ± 0.32	1.72 ± 0.36	3.63 ± 0.57	2.16 ± 0.53	3.82 ± 0.65	1.59 ± 0.63	2.20 ± 0.45	2.28 ± 0.51	2.64	R
HR-12-1-4-87-5-2-42	Xa21+xa13+xa5+ qDTY2.2+ qDTY24.1	4.17 ± 1.03	1.82 ± 0.42	3.12 ± 0.46	2.06 ± 0.98	4.22 ± 0.53	1.36 ± 0.68	2.77 ± 0.57	2.81 ± 0.59	2.79	R
HR-12-1-4-87-295-4-3	Xa21+xa13+xa5+ qDTY2.2+ qDTY24.1	2.97 ± 0.74	3.04 ± 0.45	3.64 ± 0.59	2.17 ± 0.17	3.09 ± 0.48	2.18 ± 0.24	2.89 ± 0.62	2.09 ± 0.32	2.76	R
HR-12-1-4-87-295-4-8	Xa21+xa13+xa5+ qDTY2.2+ qDTY24.1	2.66 ± 0.25	2.43 ± 0.75	2.87 ± 0.69	1.71 ± 0.25	4.08 ± 0.37	1.26 ± 1.08	2.60 ± 0.71	2.95 ± 0.78	2.57	R
HR-12-1-4-87-295-4-12	Xa21+xa13+xa5+ qDTY2.2+ qDTY24.1	3.58 ± 0.33	1.69 ± 0.71	2.77 ± 0.72	2.89 ± 0.39	2.60 ± 0.67	1.13 ± 0.59	2.63 ± 0.88	3.15 ± 0.47	2.56	R
HR-12-1-4-87-295-4-19	Xa21+xa13+xa5+ qDTY2.2+ qDTY24.1	3.27 ± 0.47	2.43 ± 0.49	4.42 ± 1.02	1.33 ± 0.21	3.36 ± 0.83	3.00 ± 0.38	2.75 ± 0.54	2.77 ± 0.45	2.92	R
HR-12-70-9-258-145-4-2	Xa21+xa13+xa5+ qDTY2.2+ qDTY24.1	2.98 ± 0.58	2.73 ± 0.33	3.63 ± 0.18	1.30 ± 0.39	4.42 ± 0.55	1.76 ± 0.54	2.82 ± 0.75	2.15 ± 0.31	2.72	R
HR-12-70-9-258-145-4-14	Xa21+xa13+xa5+ qDTY2.2+ qDTY24.1	1.47 ± 0.72	2.62 ± 0.73	3.62 ± 0.48	2.95 ± 0.34	3.94 ± 0.63	2.83 ± 056	2.70 ± 0.77	1.97 ± 0.63	2.76	R
HR-12-70-9-258-145-4-23	Xa21+xa13+xa5+ qDTY2.2+ qDTY24.1	3.68 ± 1.18	2.60 ± 0.98	3.77 ± 0.39	2.17 ± 0.58	3.13 ± 0.94	2.48 ± 0.38	2.80 ± 0.35	1.81 ± 0.49	2.80	R
HR-12-70-9-258-169-1-8	Xa21+xa13+xa5+ qDTY2.2+ qDTY24.1	4.02 ± 0.61	1.71 ± 0.54	3.28 ± 0.68	1.79 ± 0.96	3.17 ± 0.81	2.48 ± 0.44	3.50 ± 0.95	2.53 ± 0.42	2.81	R
HR-12-70-9-258-169-1-23	Xa21+xa13+xa5+ qDTY2.2+ qDTY24.1	3.37 ± 0.52	1.26 ± 0.72	3.46 ± 0.24	1.69 ± 0.71	2.76 ± 0.64	1.38 ± 0.31	2.17 ± 0.45	1.97 ± 0.33	2.26	R
HR-12-70-9-258-169-1-38	Xa21+xa13+xa5+ qDTY2.2+ qDTY24.1	3.72 ± 0.34	2.02 ± 0.32	3.28 ± 0.29	2.40 ± 0.77	3.48 ± 0.36	1.96 ± 0.92	2.61 ± 0.62	2.35 ± 0.68	2.73	R
HR-12-70-9-258-223-5-9	Xa21+xa13+xa5+ qDTY2.2+ qDTY24.1	1.49 ± 0.96	1.72 ± 0.45	3.63 ± 0.75	2.16 ± 0.61	3.82 ± 0.46	1.59 ± 0.56	3.30 ± 0.75	2.28 ± 0.29	2.50	R
HR-12-70-9-258-223-5-12	Xa21+xa13+xa5+ qDTY2.2+ qDTY24.1	1.17 ± 0.34	1.82 ± 0.14	3.12 ± 0.61	2.06 ± 0.84	4.22 ± 0.59	1.36 ± 0.37	2.77 ± 0.67	2.81 ± .60	2.42	R
HUR-917-15-2-2-1 (Donor)	Xa21+xa13+xa5+Xa4	3.07 ± 0.39	1.96 ± 0.33	3.44 ± 0.47	2.03 ± 0.39	3.07 ± 0.67	0.95 ± 0.68	2.69 ± 0.27	1.63 ± 0.27	2.36	R
HUR-917 (RP)	xa21+Xa13+Xa5	14.62 ± 1.25	8.67 ± 1.08	15.47 ± 1.98	11.09 ± 1.28	14.90 ± 0.97	8.78 ± 1.21	10.17 ± 1.69	14.98 ± 1.04	12.34	S

R, resistant; MR, moderately resistant; S, susceptible; MLL, mean lesion length in centimeters.

### 3.2 MAB based pyramiding of drought-tolerant and BB resistance genes in HUR 917

The F_1_ population (60 plants) derived from the cross HUR 917 × DRR Dhan 42 was evaluated for hybridity using gene-linked molecular markers, namely, RM279 (~2.88 cM; linked to *qDTY2*.2), RM518 (~2.02 cM; linked to *qDTY4*.1) (Swamy et al., 2013; Sandhu and Kumar, 2017), pTA-248 (~0.2 cM from *Xa21*), xa13prom (0.0 cM from *xa13*), and *xa5* (0.4 cM from *xa5*) (**Table 2**). Based on marker validation, four true F_1_ hybrids were identified and backcrossed with the RP, HUR-917-15-2-2-1, to generate the BC_1_F_1_ population. These progenies were subjected to foreground selection using target gene-linked markers and evaluated for agro-morphological similarity with the RP.

**Figure 2:**
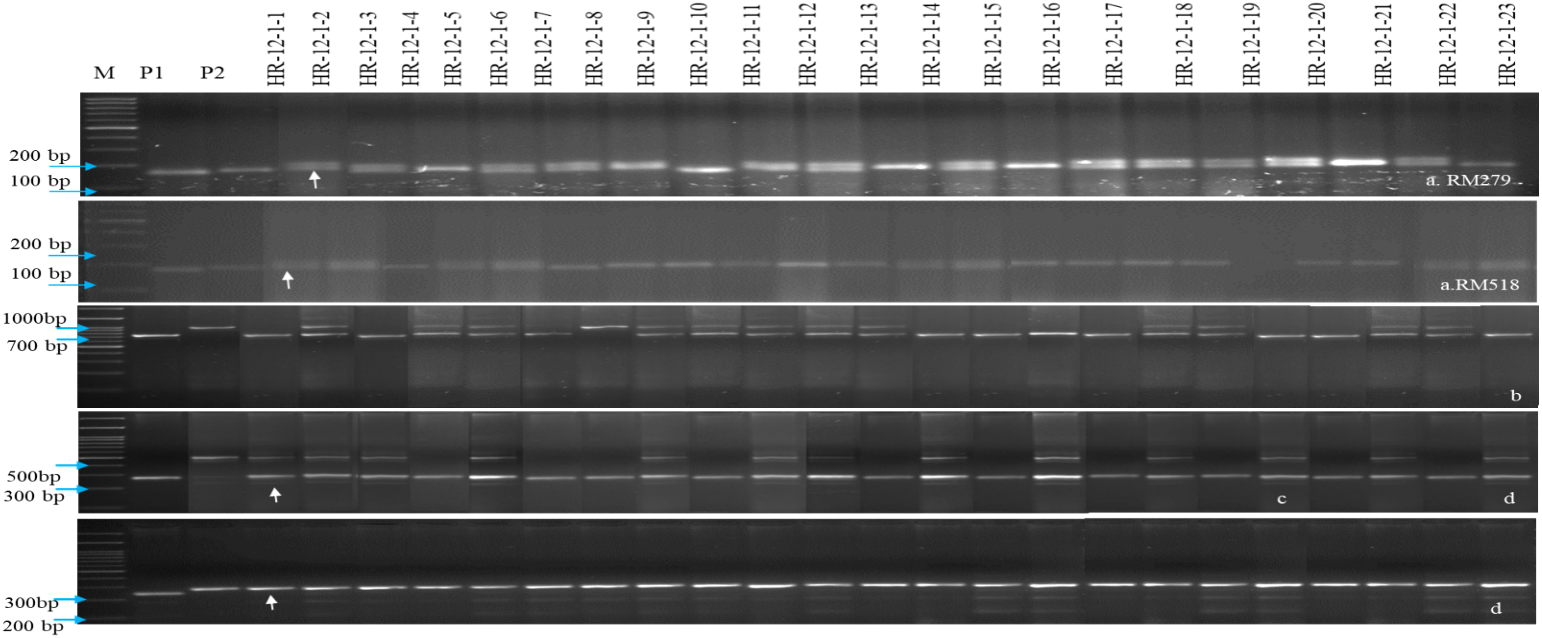
PCR amplification of tolerance/resistance gene(s) in BC_2_F_1_. **(A)** Amplicons of the *qDTY_2.2 and_ qDTY_4.1_* using RM279 and RM518 primer. **(B)** Amplicons of the *Xa21* using pTA248 primer. **(C)** Amplicons of the *xal3* using xal3prom primer. **(D)** Amplicons of the *xa5* using RM122 primer; M, marker; P1, donor parent (DRR Dhan 42 and HUR-917-15-2-2-1); P2, recurrent parent (HUR 917). Lanes 4–23 represent BC_2_F_1_ plants; vertical arrows indicate positive plants homozygous/heterozygotes for all targeted genes.

**Figure 3:**
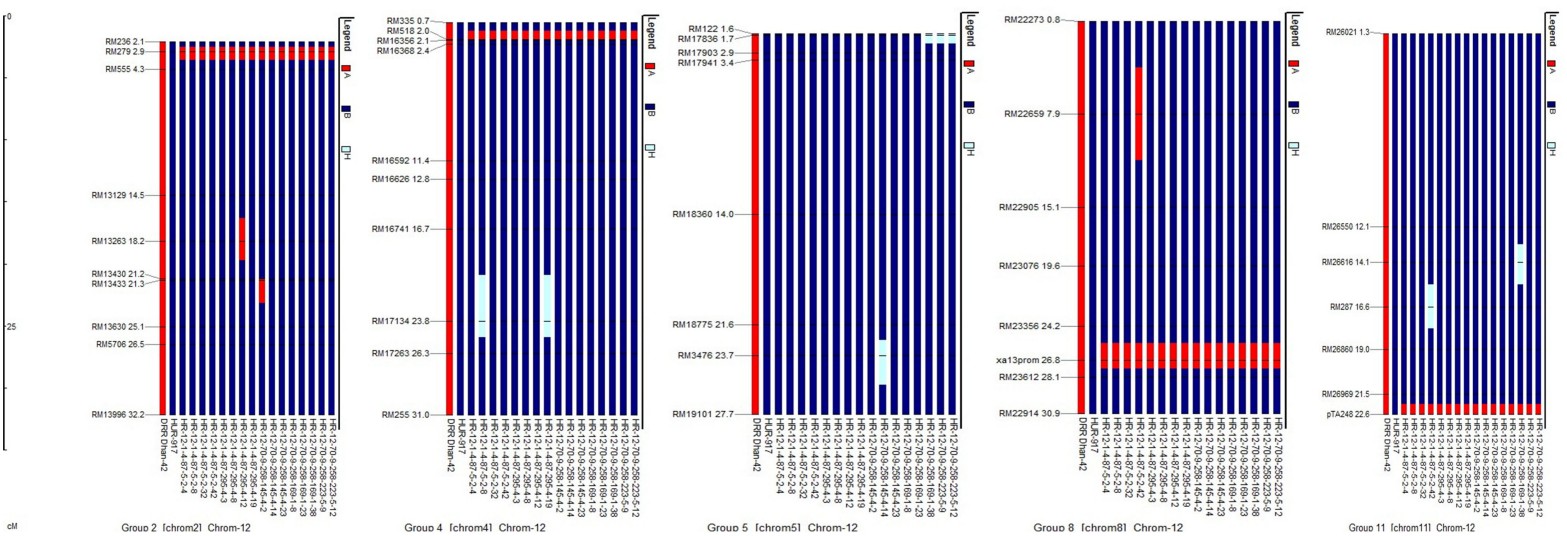
Graphical representation of genomic contributions from parents in NILs derived from the cross between HUR-917 (RP) and DRR Dhan 42 (donor), NILs: 1–16, A represents RP, B denotes donor, and H indicates heterozygosity; Chromosomes 2, 4, 5, 8, and 11 harbor *qDTY2*.2, *qDTY4*.1, R genes, *xa5, xa13*, and *Xa21*, respectively.

Out of 240 BC_1_F_1_ plants, 14 individuals were identified as heterozygous for the target loci (*qDTY2.2 + qDTY4.1 + Xa21 + xa13 + xa5*), exhibiting recurrent parent genome (RPG) recovery ranging from 67.40% to 79.57%, along with desirable phenotypic attributes, including moderate resistance (MR) to disease. These plants recorded a PDI ranging from 10.22 ± 0.236 to 32.48 ± 0.085 and an area under disease progress curve (AUDPC) between 200.54 and 483.72 (**Table 3**; **Figure 2**; **Supplementary Table 4**). Among them, five superior BC_1_F_1_ plants with >75% RPG recovery and favorable phenome were advanced to BC_2_F_1_, generating 250 seeds (**Table 3**; **Figure 3**).

**Figure 4:**
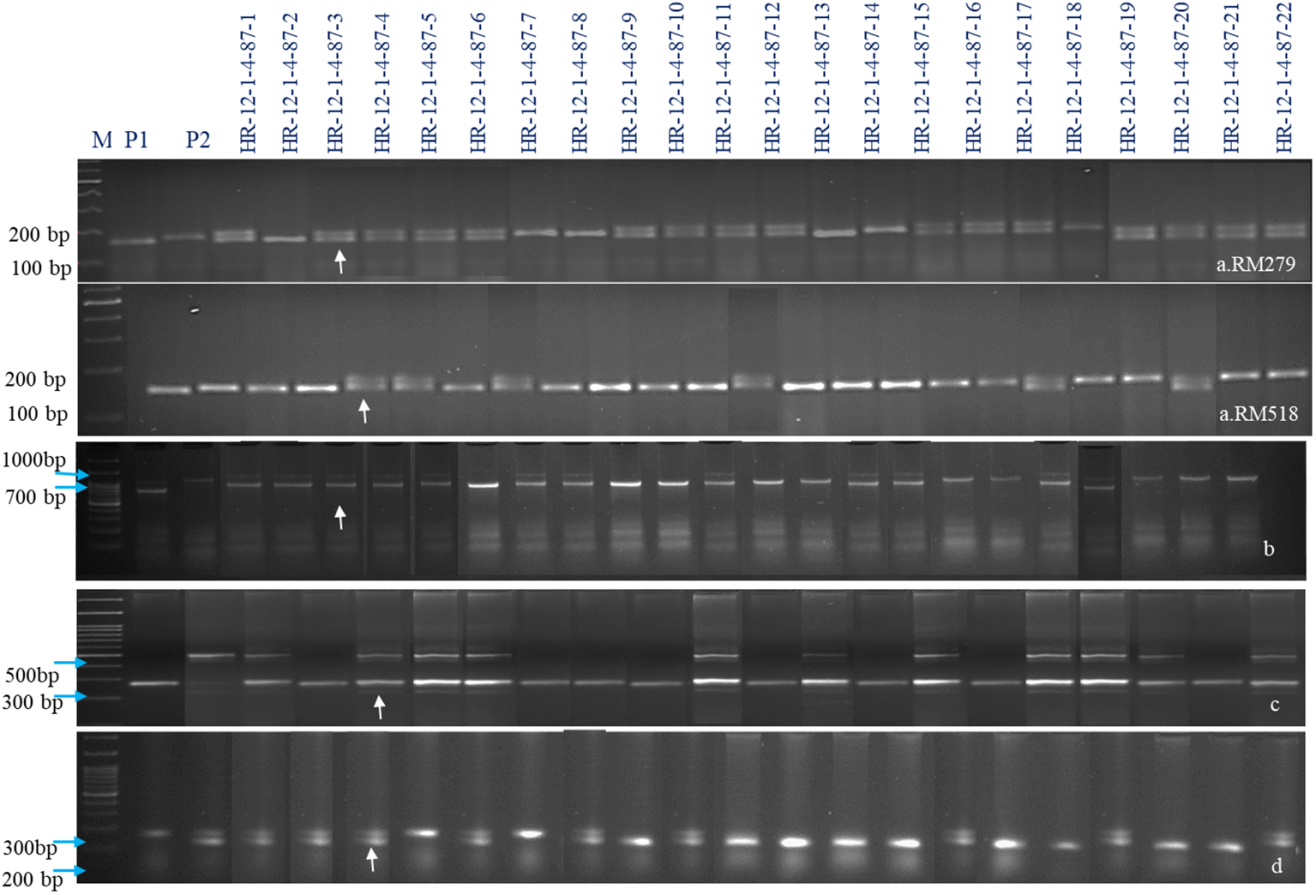
PCR amplification of tolerance/resistance gene(s) in BC_3_F_1_. **(A)** Amplicons of the *qDTY_2.2_*and*qDTY_4.1_* using RM279 and RM518 primer. **(B)** Amplicons of the *Xa21* using pTA248 primer. **(C)** Amplicons of the *xal3* using xal3prom primer. **(D)** Amplicons of the *xa5* using RM122 primer; M, marker; P1, donor parent (DRR Dhan 42 and HUR-917-15-2-2-1); P2, recurrent parent (HUR 917). Lanes 4–22 represent BC_3_F_1_ plants; vertical arrows indicate positive plants homozygous/heterozygotes for all targeted genes.

In the BC_2_F_1_ generation, eight selected plants carrying the target gene combinations showed RPG recovery of 78.57% to 87.56% and improved phenotypic performance, with PDI values of 6.05 ± 0.227 to 12.69 ± 0.328 and AUDPC ranging from 89.82 to 194.56 (MR reaction). These plants were further backcrossed with the RP to produce BC_3_F_1_ seeds (540 seeds). In the BC_3_F_1_ generation, two plants exhibiting RPG recovery between 82.65% and 89.25% along with desirable phenotypic expression (PDI: 2.48 ± 0.624 to 18.24 ± 0.225; AUDPC: 72.24–178.25; MR) were selfed to develop NILs (**Table 3**; **Figure 4**; **Supplementary Table 4**).

**Figure 5:**
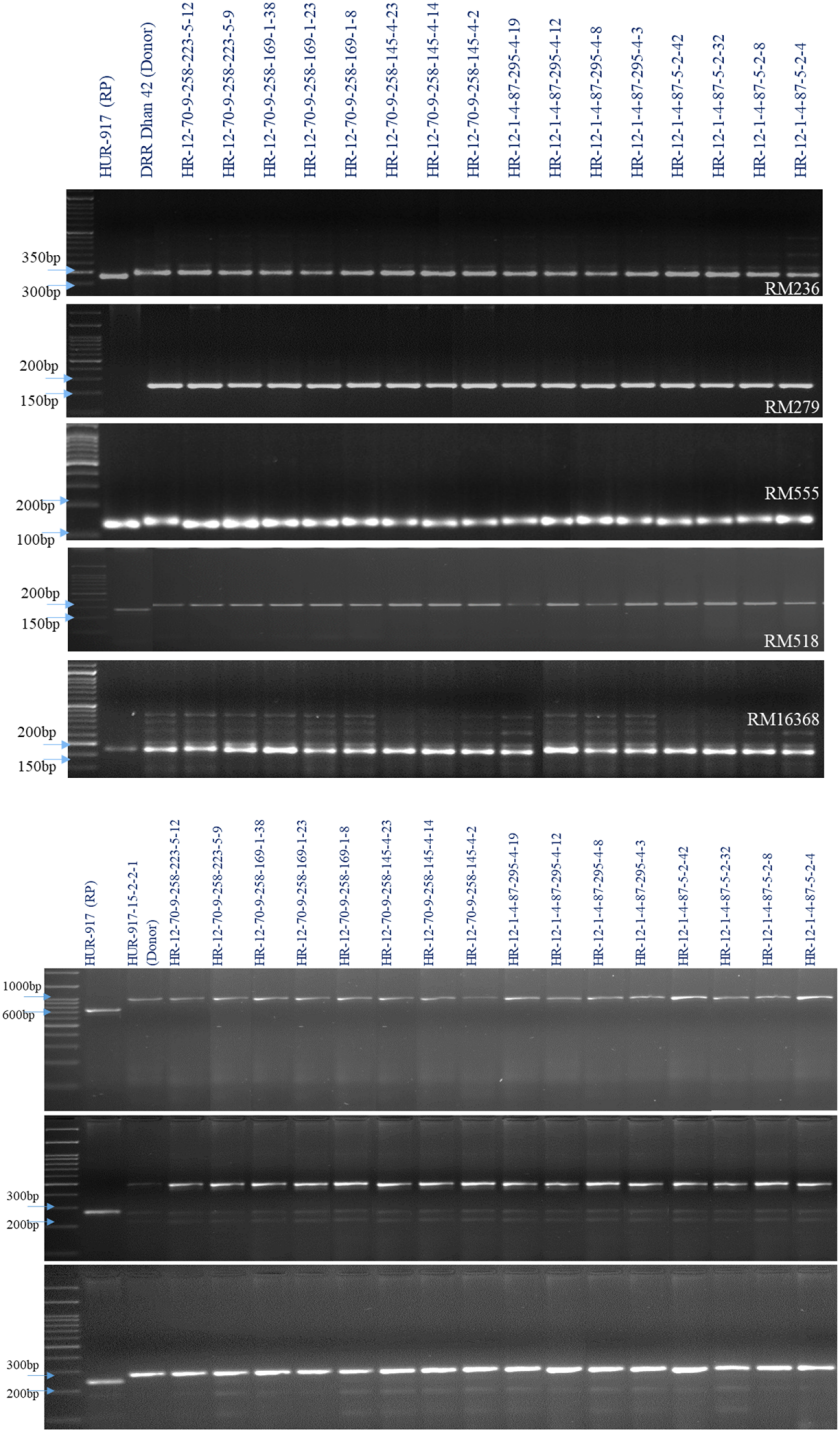
PCR amplification of tolerance/resistance gene(s) in BC_3_F_3_. **(A)** Amplicons of the *qDTY_2.2_*and*qDTY_4.1_* using RM279-RM555 and RM518-RM16368 primer. **(B)** Amplicons of the *Xa21* using pTA248 primer. **(C)** Amplicons of the *xal3* using xal3prom primer. **(D)** Amplicons of the *xa5* using RM122 primer; M, marker; P1, donor parent (DRR Dhan 42 and HUR-917-15-2-2-1); P2, recurrent parent (HUR 917). Lanes 4–19 represent BC_3_F_3_ plants; vertical arrows indicate positive plants homozygous/heterozygotes for all targeted genes.

Background selection (BS) across generations indicated a progressive reduction in heterozygosity for SSR markers, with values of 11.5%, 7.8%, and 4.2% in BC_1_F_1_, BC_2_F_1_, and BC_3_F_1_, respectively, which further decreased to 1.5% in BC_3_F_3_. Foreground screening (FS) of 1,028 BC_3_F_2_ plants identified 16 individuals homozygous for all target genes (*qDTY2.2 + qDTY4.1 + Xa21 + xa13 + xa5*) (**Figure 5**; **Supplementary Table 4**). Background analysis using 98 polymorphic SSR markers revealed >90% RPG recovery in these selected lines, including HR-12-1-4-87-5-2-4, HR-12-1-4-87-5-2-8, HR-12-1-4-87-5-2-32, HR-12-1-4-87-5-2-42, HR-12-1-4-87-295-4-3, HR-12-1-4-87-295-4-8, HR-12-1-4-87-295-4-12, HR-12-1-4-87-295-4-19, HR-12-70-9-258-145-4-2, HR-12-70-9-258-145-4-14, HR-12-70-9-258-145-4-23, HR-12-70-9-258-169-1-8, HR-12-70-9-258-169-1-23, HR-12-70-9-258-169-1-38, HR-12-70-9-258-223-5-9, and HR-12-70-9-258-223-5-12 (**Table 3**; **Supplementary Table 4**; **Figure 5**; **Supplementary Figures 2**, **3**).

**Figure 6:**
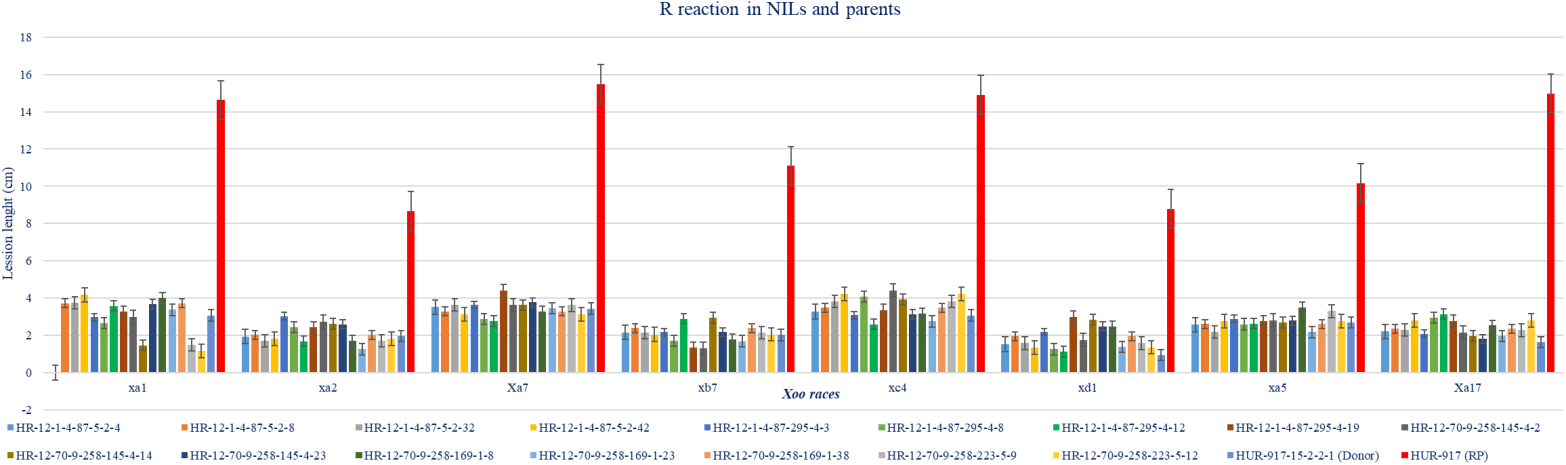
Extent of BB resistance in parents and derivative NILs (BC_3_F_3_) inoculated with 8 virulent *Xoo* isolates after 21 days of inoculation.

These 16 BC_3_F_2_ lines were advanced to the BC_3_F_3_ generation for stabilization and selection of superior NILs combining drought tolerance and BB resistance. Phenotypic evaluation under stress conditions confirmed enhanced drought tolerance (**Table 5**) and stable BB resistance, with PDI values ranging from 1.13 ± 0.59 to 4.42 ± 1.02 and AUDPC between 56.35 and 92.05 (**Table 4**; **Figure 6**; **Supplementary Table 5**).

**Table 5:** ANOVA of parents and BC_3_F_3_ and BC_3_F_4_ (pooled) derivatives of HUR917/DRR Dhan 42 for morpho-agronomical traits.

Source	Irrigated condition			Drought condition (ROS)		
	Replication	Genotype	Residual	Replication	Genotype	Residual
DF	2	16	32	2	16	32
DFF	4.05	34.58***	6.095	15.57	39.35***	4.82
DM	10.24	37.251***	4.319	26.796	37.509***	4.071
PH	8.57	27.211***	4.1623	18.29	45.15	24.02
NETPP	0.72	2.62**	0.70261	0.074	2.48***	0.89
PL	4.26	5.2770***	1.1658	18.68	2.71**	1.03
SF	0.13	9.08***	1.4345	429.42	64.25**	21.13
GYPP	0.13	4.043***	0.4516	33.294	2.560**	0.630
TW	2.526	20.211***	0.7437	0.0146	21.25***	0.0398
KL (mm)	0.17	0.425***	0.03	0.04	0.836***	0.02
KB (mm)	0.36	0.02***	0.01	0.48	0.022***	0.05
L/B ratio	0.24	0.215***	0.04	0.28	0.235***	0.02
HRR (%)	0.11	55.896***	0.47	0.17	56.698***	0.32
AC%	0.08	5.480*	0.08	0.32	4.458*	0.06
GC	0.05	184.365***	0.24	0.28	198.708***	0.24
Aroma	0.01	0.904***	0.01	0.03	0.632***	0.01
LR	0.00	0.00	0.00	0.2222	3.96***	0.2222
CC	19.574	130.205***	1.417	47.614	57.03***	0.767
PC	8.7963	27.4564***	1.1492	51.52	592.36***	0.72
PDI with SD (%) at 14 DAI	0.03	98.257***	0.02	0.01	136.221***	0.07
PDI with SD (%) at 21 DAI	0.44	41.742***	0.04	0.23	248.123***	0.04
PDI with SD (%) 28 DAI	0.02	72.865***	0.08	0.2	425.408***	0.02
AUD PC	0.53	75.236.199***	1.22	0.56	92.362***	1.05
GR%	0.02	114.254***	0.06	0.08	138.268***	0.05

*, **, and *** significant at *p* ≤ 0.05, *p* ≤ 0.01, and *p* ≤ 0.001 probability level, respectively.

DFF, days to 50% flowering; DM, days to maturity; PH, plant height; NETPP, number of effective tillers per plant; PL, panicle length; SF, spikelet fertility; GYPP, grain yield per plant; TW, test weight; KL, kernel length; KB, kernel breadth; KLBR, kernel length/breadth ratio; HRR, head rice recovery; AC, amylose content; GC, gelatinization content; PDI, percent disease index; DAI, days after inoculation; AUDPC, area under disease progress curve; GR, genome recovery; LR, leaf rolling; CC, chlorophyll content; PC, proline content.

Phenotypic assessment across backcross generations indicated minimal selection differential (Δ*d*) for key product profile traits, including DFF (0.9–2.08), PH (1.08–2.36), grain L/B ratio (0.06–0.14), HRR (0.42–1.57), and aroma (0.0), suggesting the effective recovery of the RP phenotype (**Supplementary Table 4**).

The improved NILs carrying the pyramided genes (*qDTY2.2 + qDTY4.1 + Xa21 + xa13 + xa5*) exhibited strong drought-responsive traits, such as low leaf rolling score (1.0), higher chlorophyll content (44.33–55.00), and elevated proline accumulation (65.44–80.00) (**Table 5**; **Supplementary Table 7**). These lines also demonstrated broad-spectrum BB resistance, with lesion length and PDI values ranging from 1.13 ± 0.59 to 4.42 ± 1.02 and 2.34 ± 0.84 to 4.38 ± 1.14, respectively, and AUDPC values between 56.35 and 92.05 (**Table 4**; **Figure 6**).

Grain quality analysis revealed that the NILs closely resembled the RP, with negligible selection differential for grain L/B ratio (Δ*d* = 0.14) and HRR (Δ*d* = 0.59), along with comparable cooking quality and palatability (**Table 5**; **Supplementary Table 4**). Genetic similarity analysis based on 98 SSR markers indicated similarity coefficients ranging from 0.91 to 0.95 between the NILs and the RP (**Supplementary Figure 2**). Cluster analysis grouped all 18 genotypes into two major clusters: Cluster I-A comprising only DRR Dhan 42 (similarity coefficient, 0.77) and Cluster II containing HUR 917 and all 16 NILs, with maximum similarity (0.94) observed between HUR 917 and lines HR-12-1-4-87-5-2-4, HR-12-1-4-87-295-4-8, and HR-12-70-9-258-145-4-23.

**Figure 7:**
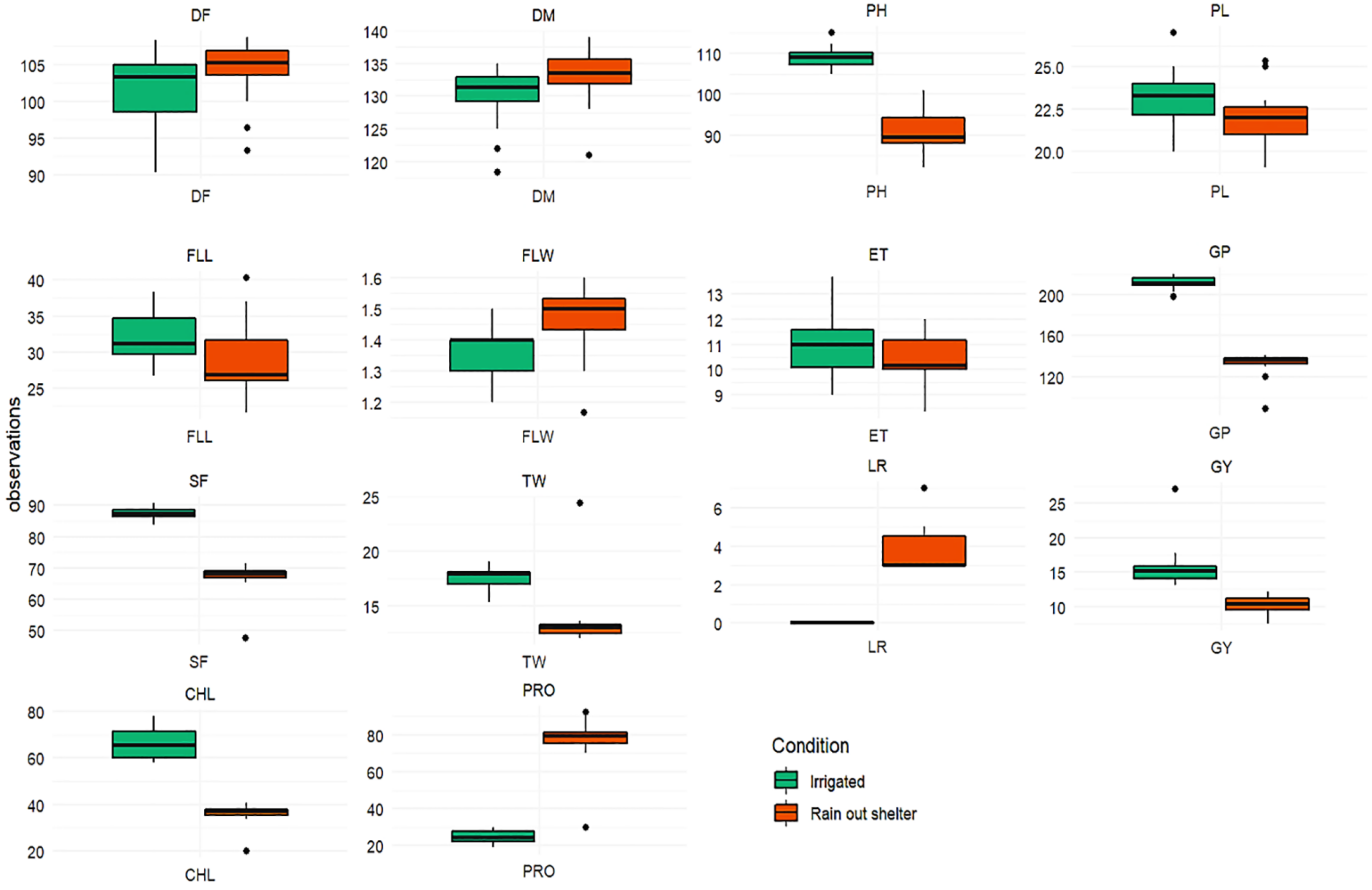
Comparative analysis of agro-morphological traits in _BC3F4_ under irrigated and rainout shelter conditions.

Overall, the majority of NILs exhibited high phenotypic resemblance to HUR 917 in terms of yield, grain quality, and adaptability traits (**Table 5**). The selected NILs with >90% RPG recovery successfully achieved the desired product profile of the RP while incorporating drought tolerance and BB resistance (**Figure 7**). Notably, lines HR-12-1-4-87-5-2-4, HR-12-1-4-87-5-2-8, HR-12-1-4-87-295-4-3, HR-12-1-4-87-295-4-12, HR-12-1-4-87-295-4-19, and HR-12-70-9-258-223-5–12 demonstrated yield performance, TW, HRR, AC, and survivability comparable to the RP under both stress and non-stress conditions (**Supplementary Table 7**). Quality evaluation further confirmed that these NILs maintained desirable grain characteristics, including HRR (62.0%–66.3%) and intermediate AC (22.1%–24.2%), with no significant differences in aroma and palatability compared to the RP (**Table 5**; **Supplementary Table 7**).

### 3.3 Genome introgression in carrier and non-carrier chromosomes

The success of the trait improvement strategy relies upon the extent of the RP genome recovery in the resulting products while retaining originality of the RP. The results revealed that all NILs attained a high level of RPG recovery, averaging 92.02%, with >90% genomic contribution from chromosomes 1, 3, 5, 6, 7, 10, and 12 (**Figure 7**; **Supplementary Table 4**; **Supplementary Figure 3**). Complete restoration of the RPG was recorded for chromosomes 5, 6, 7, and 9 across all NILs. The drought-associated QTLs, *qDTY2*.2 (chromosome 2) and *qDTY4*.1 (chromosome 4), were successfully introgressed with minimal LD. For *qDTY4*.1, LD was confined to 1.3 Mb upstream and 0.1 Mb downstream, flanked by markers RM235 and RM16356, respectively. Similarly, LD around *qDTY2*.2 extended up to 0.5 Mb upstream (RM236) and 1.4 Mb downstream (RM555) (**Supplementary Figure 4**). The BB resistance genes *xa5, xa13*, and *Xa21*, located on chromosomes 5, 8, and 11, respectively, were also effectively pyramided with negligible donor genome segments from HUR-917-15-2-2-1. Recombinant analysis indicated that LD for *xa5* was restricted to 0.1 Mb downstream (RM17836). In contrast, LD surrounding *xa13* extended up to 2.6 Mb upstream and 1.9 Mb downstream, flanked by RM23356 and RM23612, while LD for *Xa21* was limited to 1.1 Mb upstream with marker RM26969 (**Figure 7**; **Supplementary Figure 4**). Analysis using linked markers confirmed successful introgression of both drought tolerance QTLs and BB resistance genes in all improved NILs, each possessing more than 90% RP genome recovery (**Figure 7**).

Further validation using marker RM122 confirmed the presence of *xa5* in all 16 NILs with high RPG (>90%) (**Figure 7**). Similarly, the xa13prom marker profile verified successful introgression of *xa13* across all NILs (1–16), with RPG recovery exceeding 87.5% in most lines, except HR-12-1-4-87-5-2-42, which showed 75% RP genome recovery on chromosome 8. All NILs carrying *Xa21* exhibited more than 80% RP genome recovery along with desirable phenotypic characteristics (**Table 5**; **Figure 7**; **Supplementary Table 7**).

In the BC_3_F_3_ generation, background marker analysis revealed that, on average, 91.25 markers (93.11%) were homozygous for RP alleles, 1.50 markers (1.53%) remained heterozygous, and 5.25 markers (5.36%) were homozygous for donor alleles (**Supplementary Table 4**). The pyramided lines, HR-12-1-4-87-5-2-4, HR-12-1-4-87-295-4-8, HR-12-70-9-258-145-4-23 (93.0%), HR-12-1-4-87-5-2-32, HR-12-70-9-258-145-4-14 (92.51%), and HR-12-1-4-87-5-2-42, HR-12-1-4-87-295-4-3, HR-12-70-9-258-169-1-23, and HR-12-70-9-258-223-5-9 (92.02%) demonstrated substantial RPG recovery along with the presence of all target genes and desirable morphological traits (**Supplementary Table 4**; **Supplementary Figure 3**; **Table 5**; **Supplementary Table 7**; **Supplementary Figures 5**, **6**).

### 3.4 Screening of NILs for drought tolerance

**Figure 8:**
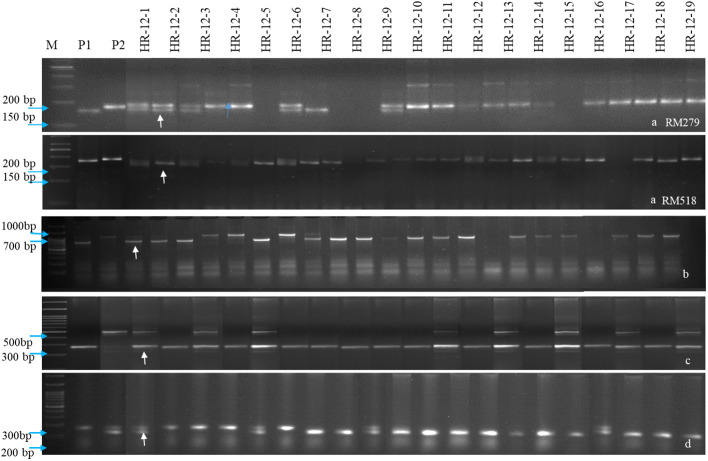
PCR amplification of tolerance/resistance gene(s) in BC_1_F_1_. **(A)** Amplicons of the *qDTY_2.2_*and *qDTY_4.1_* using RM279 and RM518 primer. **(B)** Amplicons of the *Xa21* using pTA248 primer. **(C)** Amplicons of the *xal3* using xal3prom primer. **(D)** Amplicons of the *xa5* using RM122 primer; M, marker; P1, donor parent (DRR Dhan 42 and HUR-917-15-2-2-1); P2, recurrent parent (HUR 917). Lanes 4–19 represent BC_1_F_1_ plants; vertical arrows indicate positive plants homozygous/heterozygotes for all targeted genes.

The NILs retained similarity with the RP for most agronomic traits, maintaining the desired product profile (**Table 6**; **Figure 8**). Under non-stress conditions, the majority of NILs showed comparable flowering duration, along with equal or higher grain yield per plant, TW, moderate AC, and improved HRR relative to the RP (**Supplementary Table 7**). However, under drought stress, most of NILs exhibited delayed flowering (by 5 -7 days), reduction in plant height (about 10 cm), decline in grain yield per plant (40% -50%), decreased spikelet fertility (15% -20% in NILs compared to ~40% in RP), and reduction in test weight (10% -15% in RP and most NILs). In contrast, AC and HRR remained unchanged in both NILs and the RP. Panicle length and FLL were reduced under stress, whereas FLW remained stable across environments. Despite the negative impact of drought on yield, mainly through reduced SF and grain number per panicle, NILs maintained relatively higher values for these traits compared to the RP, resulting in improved performance under stress. Notably, no yield penalty was observed in pyramided lines under irrigated conditions. Selected NILs, including HR-12-1-4-87-5-2-4, HR-12-1-4-87-5-2-8, HR-12-1-4-87-5-2-32, HR-12-1-4-87-295-4-19, HR-12-70-9-258-169-1-8, and HR-12-70-9-258-223-5-12, with more than 90% RPG recovery, consistently expressed key agronomic traits such as DFF, SF, GYPP, TW, AC, and HRR under both stress and non-stress conditions. These results demonstrate that NILs carrying *qDTY2*.2 and *qDTY4*.1 confer a yield advantage over RP under moderate drought stress without compromising performance under normal conditions. Moreover, aroma in all NILs and RP remained intact in both stressed and non-stressed conditions, further supporting the lower volatility of aroma in the non-basmati short grain genotype in an extreme environment. The developed NILs retained the grain and cooking quality traits of HUR-917, while also incorporating drought tolerance and BB resistance.

**Table 6:** Comparison of agronomic and grain quality traits of the NILs *vis-à-vis* parents in the BC_3_F_3_ and BC_3_F_4_ generation (pooled) under stressed (S) and non-stressed (NS) conditions.

Trait	Environment	RP	DP	NILs		RUs	CV	LSD
				Mean	Range			
DFF	NS	112	96	109.4	102–114.3	−4.20	2.33	7.3
	S	117	101	114	107–119		1.89	6.17
DM	NS	138	123	135.4	128–140.3	−1.95	1.88	7.3
	S	141	126	138.04	131–142		1.49	5.89
PH (cm)	NS	110.67	117.33	110	106–114	9.62	2.07	7.3
	S	100	106	99.42	96.33–102		1.99	5.73
PL (cm)	NS	24.37	26.6	23.6	19.6–25.7	5.30	3.97	2.71
	S	23.53	23.6	22.35	20–23.70		8.74	5.65
NETPP	NS	11.3	10.67	11.3	9.0–13.7	8.58	10.27	3.33
	S	10.33	10	10.33	9.0–12.0		6.38	1.89
NGPP	NS	213	182	209.2	193–224	18.93	2.76	16.53
	S	151.66	173.33	169.6	155.33–185.33		2.77	13.48
SFP (%)	NS	91.42	87.11	89.2	85.05–91.7	11.39	2.62	6.73
	S	69.78	84.32	79.04	76.90–82.30		2.79	6.34
TW (g)	NS	14.43	27.24	14.9	13.42–17.3	13.89	3.55	1.6
	S	13.08	24.2	12.83	12.37–13.59		3.54	2.03
GYPP (g)	NS	17.05	22.25	18.3	17.29–20.0	27.27	4.86	2.06
	S	10.86	15.91	13.31	12.40–14.15		6.20	2.37
LR	NS	0	0	0	0	0.00	0	0
	S	3	1	1	1.0–1.0		1.31	4.2
CC	NS	65.67	64.67	70.9	61.66–82.0	31.16	2.91	5.92
	S	38.67	47.67	48.81	44.33–55.00		10.41	14.42
PC	NS	25.67	27.33	28.5	24.66–32.7	−152.88	5.51	4.46
	S	41.33	63.33	72.07	65.44–80.00		9.92	19.92
HRR (%)	NS	66.74	59.98	65.1	63.86–66.4	1.87	1.55	2.91
	S	63.59	62.21	63.88	63.23–69.55		0.71	1.3
KL (mm)	NS	4.87	5.24	4.8	4.52–5.1	−4.58	2.005	0.27
	S	4.88	5.25	5.02	4.87–5.20		1.02	0.14
KB (mm)	NS	1.55	1.89	1.6	1.55–1.7	3.13	1.91	0.09
	S	1.51	1.9	1.55	1.51–1.60		1.16	0.05
L/B ratio	NS	2.89	2.39	2.5	2.69–3.02	−29.60	2.91	0.24
	S	2.90	2.76	3.08	2.68–3.42		1.55	0.14
KLAC	NS	7.9	8.23	2.5	7.6–8.0	−214.40	1.39	0.31
	S	7.63	8.03	7.86	7.70–8.11		1.07	0.24
ER	NS	1.62	1.57	1.6	1.58–1.7	1.88	2.02	0.09
	S	1.56	1.53	1.57	1.53–1.61		1.14	0.05
AC (%)	NS	23.27	22.69	23.2	22.23–23.9	−0.82	1.67	1.12
	S	23.09	22.89	23.01	22.28–23.49		1.45	0.96
GC	NS	44.66	43.33	40.8	36.66–43.7	−0.86	3.98	4.7
	S	41	40.67	41.15	39.00–44.67		2.59	3.06
Aroma	NS	2	0	2	2.0–2.0	0.00	1.32	7.17
	S	2	0	2	2.00–2.00		1.69	9.22

DFF, days to 50% flowering; DM, days to maturity; PH, plant height; NETPP, number of effective tillers per plant; PL, panicle length; NGPP, number of grains per panicle; SFP, spikelet fertility percentage; TW, test weight; GYPP, grain yield per plant; KL, kernel length; KB, kernel breadth; KLBR, kernel length/breadth ratio; HRR, head rice recovery; AC, amylose content; GC, gelatinization content; PDI, percent disease index; DAI, days after inoculation; AUDPC, area under disease progress curve; GR, genome recovery; LR, leaf rolling; CC, chlorophyll content; PC, proline content.

### 3.5 Physiological responses of drought-tolerant QTLs in NILs

Physiological responses further supported enhanced drought tolerance in NILs. Leaf rolling was more pronounced under stress, while chlorophyll content declined compared to well-watered conditions. However, NILs maintained relatively higher chlorophyll content (average 44.82%) than the RP, indicating better stress tolerance. Greater variability among NILs was observed for physiological traits such as chlorophyll retention and proline accumulation under drought. Maximum chlorophyll content was recorded under control conditions (60–79 mg g^-^¹), particularly in genotypes carrying *qDTY2*.2 and *qDTY4*.1, followed by a decline under moisture stress. Proline accumulation, which plays a key role in osmotic adjustment under drought, increased significantly in all NILs and the donor parent. NILs exhibited substantially higher proline content, averaging 77.06 mg g^-^¹ compared to 30 mg g^-^¹ in the RP. Several NILs, including HR-12-1-4-87-5-2-4, HR-12-1-4-87-5-2-8, HR-12-1-4-87-5-2-32, HR-12-1-4-87-5-2-42, HR-12-1-4-87-295-4-3, HR-12-1-4-87-295-4-8, HR-12-1-4-87-295-4-19, HR-12-70-9-258-169-1-8, HR-12-70-9-258-169-1-23, HR-12-70-9-258-169-1-38, HR-12-70-9-258-223-5-9, and HR-12-70-9-258-223-5-12, recorded proline levels exceeding 80 mg g^-^¹ under drought stress. Overall, the results confirm that introgression of *qDTY2*.2 and *qDTY4*.1 enhances drought tolerance and yield stability without introducing undesirable effects, thereby demonstrating their effectiveness in improving productivity under moisture-limited environments.

### 3.6 Bioassay of NILs for bacterial blight resistance

Bioassay evaluation of BC_3_F_3_ and BC_3_F_4_ NILs indicated a high level of resistance to BB, with lesion length (LL) ranging from 1.13 ± 0.59 cm to 4.42 ± 1.02 cm and PDI ranging from 2.34 ± 0.84 to 4.38 ± 1.14, comparable to the donor parent (**Table 4**; **Figure 6**; **Supplementary Figure 5**). All 16 NILs that were homozygous for the target genes/QTLs demonstrated superior performance over the RP under moderate drought stress and exhibited broad-spectrum resistance against eight virulent *Xoo* pathotypes prevalent in eastern India. These NILs possessed fixed alleles of *qDTY2*.2, *qDTY4*.1, and resistance genes *Xa21, xa13*, and *xa5*, with RPG recovery ranging from 90.51% to 93.0%. The observed LL values corresponded to a resistant reaction (disease score of 1).

## 4 Discussion

The effects of climate change are increasingly manifested through irregular rainfall patterns, which intensify the susceptibility of rice cultivation to drought stress. Enhancing the intrinsic drought tolerance of widely cultivated varieties has therefore become essential for improving climate resilience. HUR-917, an aromatic rice cultivar with high market value, is well suited to lowland ecosystems but remains vulnerable to water deficit conditions. In this study, an effort was made to improve its adaptability to reproductive stage drought stress (RSDS) along with enhanced sustainability under BB severity through targeted introgression, thereby strengthening its suitability for rainfed environments.

Moisture stress continues to be a major limitation to rice productivity, particularly in rainfed systems, as it adversely affects physiological processes and key yield components. Yield decline under drought is largely associated with reduced SF, lower grain number per panicle, and impaired grain filling (Pantuwan et al., 2002; Kumar et al., 2006). In monsoon-dependent regions such as India, the impact of drought is further amplified due to erratic rainfall and increasing climatic variability, often resulting in substantial yield losses (Gautam and Bana, 2014).

The frequent occurrence of drought, coupled with the emergence of diverse and virulent *Xoo* races, poses a significant challenge for sustainable rice production in rainfed lowland ecosystem. Despite the availability of improved varieties, productivity in rainfed ecosystems remains low and unstable (Verulkar et al., 2010). Extended drought periods can also predispose crops to multiple biotic stresses, further threatening yield stability. In this context, host plant resistance offers an environmentally sustainable approach to mitigate both abiotic and biotic stresses. Previous studies have demonstrated that pyramiding of multiple resistance genes or tolerance QTLs can lead to additive or synergistic effects, thereby enhancing overall stress resilience (Singh et al., 2011; Singh R. et al., 2015). Advances in molecular breeding tools have further accelerated the development of improved cultivars, with marker-assisted breeding emerging as an efficient and precise strategy for trait introgression (Sundaram et al., 2008; Sandhu et al., 2019).

HUR-917, derived from Dehradun Basmati Selection-13, is widely appreciated for its superior grain and cooking quality in the Indo-Gangetic plains of eastern Uttar Pradesh. It is characterized by medium duration, semi-dwarf plant type, compact panicles, and desirable grain quality traits, including intermediate AC and excellent cooking quality (Singh R. P. et al., 2015). However, its susceptibility to drought limits its performance under water-scarce conditions. Given the increasing risk of water deficit due to climate change, improving drought tolerance in this variety is of considerable importance. Selected NILs, including HR-12-1-4-87-5-2-4, HR-12-1-4-87-5-2-8, HR-12-1-4-87-5-2-32, HR-12-1-4-87-295-4-19, HR-12-70-9-258-169-1-8, and HR-12-70-9-258-223-5-12, with more than 90% RPG recovery, consistently expressed key agronomic traits such as DFF, SF, GYPP, TW, AC, and HRR under both stress and non-stress conditions. These results demonstrate that NILs carrying *qDTY2*.2 and *qDTY4*.1 confer a yield advantage over RP under moderate drought stress without compromising performance under normal conditions. Furthermore, aroma in all NILs and RP remained intact in both stressed and non-stressed conditions, further supporting the lower volatility of aroma in the no-basmati short grain genotype in an extreme environment. The generated NILs insulated with drought tolerance and BB resistance while retained the grain and cooking quality characteristics of HUR-917, while also incorporating drought tolerance and BB resistance.

Several QTLs associated with drought tolerance have been identified and validated in rice, and their deployment through marker-assisted backcrossing has proven effective in improving complex traits (Vikram et al., 2011; Dwivedi et al., 2021; Dhawan et al., 2021). In the present study, two major drought-tolerant QTLs, *qDTY2*.2 and *qDTY4*.1, known for their consistent performance in enhancing yield under drought were introgressed into HUR-917 along with BB resistance genes (*Xa21, xa13*, and *xa5*) using a marker-assisted breeding approach. The use of tightly linked SSR markers enabled precise foreground selection, while background selection facilitated rapid recovery of the RPG.

The developed NILs exhibited high RPG (>90%) along with successful introgression of target QTLs and resistance genes. Under drought conditions, these lines showed typical stress responses, including delayed flowering (5–7 days), reduced plant height (~10 cm), and decreased PL and FLL, while FLW remained stable (Moonmoon and Islam, 2017).

Physiological responses such as increased leaf rolling, reduced chlorophyll content, and enhanced proline accumulation indicated effective adaptation to moisture stress. The elevated proline levels observed in NILs are consistent with its role in osmotic adjustment and stress tolerance (Kumar et al., 2008; Caballero et al., 2005).

Despite the adverse effects of drought, the introgressed lines maintained higher SF and grain number per panicle compared to the RP, resulting in improved yield performance under stress. Importantly, no yield penalty was observed under non-stress conditions, indicating that the introgressed QTLs did not negatively affect productivity. Most of the NILs demonstrated superior yield under both drought and irrigated environments, confirming the effectiveness of the introgression strategy and the positive contribution of *qDTY2*.2 and *qDTY4*.1 to yield stability (Bhukya et al., 2020; Swamy et al., 2013) (DS 2022-23).

A major challenge in this study was the transfer of drought tolerance into an aromatic background without compromising grain quality. However, detailed quality assessment revealed that the NILs largely retained the characteristic grain and cooking quality traits of HUR-917 under both stress and non-stress conditions. Although minor variations such as slight reductions in TW and increased chalkiness were observed under drought, overall quality remained acceptable, demonstrating the success of MABB in preserving essential traits (Dhawan et al., 2021; Muthu et al., 2020) (DS-2022-23).

The bioassay evaluation further confirmed the effective introgression of BB resistance genes, with NILs exhibiting strong resistance against multiple virulent *Xoo* pathotypes (Kumar et al., 2023; Ellur et al., 2016). Lines carrying combinations of two or more resistance genes showed resistance levels comparable to the donor, highlighting the advantage of gene pyramiding in achieving durable resistance (Das et al., 2018; Pradhan et al., 2019) (DS 2022-23).

Overall, the study reaffirms the effectiveness of marker-assisted gene introgression in developing improved rice lines with enhanced tolerance to multiple stresses while maintaining the desirable attributes of the RP (Singh et al., 2022; Das et al., 2022; Janaki Ramayya et al., 2021). The high level of RPG recovery achieved with a moderate number of markers further validates the efficiency of the approach. These findings demonstrate that precise and rapid development of improved cultivars with multiple trait integration is feasible, providing a strong foundation for future breeding programs aimed at enhancing climate resilience in rice.

## 5 Conclusion

The NILs developed through the introgression of drought-yield QTLs (*qDTY2*.2 and *qDTY4*.1) and BB resistance genes into the high-quality aromatic rice variety HUR-917, using integrated phenotypic and molecular approaches, exhibited no detrimental effects on grain quality or key agronomic traits. The NILs, namely, HR-12-1-4-87-5-2-4, HR-12-1-4-87-5-2-8, HR-12-1-4-87-5-2-32, HR-12-1-4-87-295-4-19, HR-12-70-9-258-169-1-8, and HR-12-70-9-258-223-5-12, with more than 90% RPG recovery, consistently maintained essential traits such as DFF, SF, GYPP, TW, AC, and HRR under both stress and non-stress environments. These improved NILs represent a significant advancement of HUR-917 by combining its superior grain quality with enhanced BB resistance and improved yield performance under drought conditions. The study demonstrates the effectiveness of marker-assisted introgression in simultaneously incorporating multiple QTLs and resistance genes, enabling precise and rapid improvement of complex traits such as drought tolerance and disease resistance. The developed NILs provide a promising alternative for cultivation in regions where HUR-917 is grown under limited soil moisture conditions, allowing reduced irrigation requirements without compromising yield and grain quality. Their development facilitates the expansion of aromatic rice cultivation into drought-prone environments. Furthermore, these introgressed lines can serve as valuable donor sources for the rapid transfer of drought tolerance and disease resistance into other elite aromatic short-grain rice varieties with minimal linkage drag.

## Data Availability

The datasets presented in this study can be found in online repositories. The names of the repository/repositories and accession number(s) can be found in the article/**Supplementary Material**.
