# Supplementary material for: Marker-assisted trait enhancement for drought tolerance and bacterial leaf blight resistance in rice cultivar HUR-917
Source: Front Plant Sci. 2026 Jul 14;17:1848543. doi: 10.3389/fpls.2026.1848543 (PMC13408217; doi:10.3389/fpls.2026.1848543)
Supplement: Supplementary file 1 [file DataSheet1.pdf]

Ttile: Marker-Assisted Trait Enhancement for Drought Tolerance and Bacterial Leaf Blight Resistance in Rice Cultivar HUR917

Authors:

Pandurang B Arsode[1,2#], Prakash Singh[3*], S.K. Singh[1], RP Singh[1], Manish Kumar[1,4], Debarchana Jena[2], Diptibala Rout[2], Tanyashree Devalaxmi[2], Somyashree Mishra[2], Vineeta Singh[2], Namrata[1,5], JL Katara[2], S. Samantaray[2], Ramlakhan Verma[2*] and Vijay Pal Bhadana[6*]

Affiliations:

[1]Department of Genetics and Plant Breeding, Institute of Agricultural Sciences, Banaras Hindu University, Varanasi, Uttar Pradesh, India 221005
[2]ICAR-Central Rice Research Institute, Cuttack, Odisha, India 753006
[3]Botanical Research Unit-BRU, Dhangain, Bikramganj (Bihar Agricultural University-BAU), Sasaram, Rohtas, Bihar - 802 212, India 802212
[4] Department of Genetics and Plant Breeding, Rajasthan Agricultural Research Institute (Sri Karan Narendra Agriculture University, Jobner), , Durgapura, Rajasthan 302018
[5] Dr.BRC Agriculture Research Station, Mandor (Agriculture University, Jodhpur), Rajasthan, India 342304
[6]ICAR-Indian Institute of Agricultual Biotechnology, Ranchi, Jharkhand 834003

*Correspondance: ram.pantvarsity@gmail.com, prakash201288@gmail.com and bhadanavijai@gmail.com (Joined correspondance)

Short title: "Marker-Assisted Improvement of popular rice cultivar HUR917

Supplementary Table 1. Similarity matrix of parental lines taken for genetic diversity analysis

Genotypes	HUR-917	DRR Dhan-42	Nagina-22	Vandana	Dhagaddeshi
HUR-917	1				
DRR Dhan-42	0.77	1			
Nagina-22	0.59	0.06	1		
Vandana	0.50	0.70	0.65	1	
Dhagaddeshi	0.17	0.33	0.33	0.50	1

Supplementary Table 2. Similarity matrix NILs

Genotype	RP	1	2	3	4	5	6	7	8	9	10	11	12	13	14	15
DRR Dhan-42	0.77															
HUR-917	1															
HR-12-1-4-87-5-2-4	0.94															
HR-12-1-4-87-5-2-8	0.91	0.97														
HR-12-1-4-87-5-2-32	0.93	0.99	0.96													
HR-12-1-4-87-5-2-42	0.86	0.92	0.89	0.91												
HR-12-1-4-87-295-4-3	0.92	0.98	0.95	0.97	0.92											
HR-12-1-4-87-295-4-8	0.94	1.00	0.97	0.99	0.92	0.98										
HR-12-1-4-87-295-4-12	0.92	0.98	0.95	0.97	0.90	0.96	0.98									
HR-12-1-4-87-295-4-19	0.91	0.97	0.96	0.96	0.90	0.95	0.97	0.97								
HR-12-70-9-258-145-4-2	0.91	0.97	0.96	0.96	0.89	0.95	0.97	0.95	0.94							
HR-12-70-9-258-145-4-14	0.92	0.98	0.95	0.97	0.90	0.96	0.98	0.96	0.95	0.95						
HR-12-70-9-258-145-4-23	0.94	1.00	0.97	0.99	0.92	0.98	1.00	0.98	0.97	0.97	0.98					
HR-12-70-9-258-169-1-8	0.92	0.98	0.95	0.97	0.90	0.96	0.98	0.96	0.95	0.95	0.96	0.98				
HR-12-70-9-258-169-1-23	0.92	0.98	0.95	0.97	0.92	1.00	0.98	0.96	0.95	0.95	0.96	0.98	0.96			
HR-12-70-9-258-169-1-38	0.90	0.96	0.93	0.95	0.88	0.94	0.96	0.94	0.93	0.93	0.94	0.96	0.94	0.94		
HR-12-70-9-258-223-5-9	0.92	0.98	0.95	0.97	0.90	0.96	0.98	0.96	0.95	0.95	0.96	0.98	0.98	0.96	0.96	
HR-12-70-9-258-223-5-12	0.91	0.97	0.94	0.96	0.91	0.95	0.97	0.95	0.94	0.94	0.95	0.97	0.95	0.95	0.97	0.97

Supplementary Table 3. A list of polymorphic SSR markers between parents HUR 917, DR Dhan 42 and imp HUR917 (HUR-917-15-2-2-1)

Chromosome	Total SSRs	Polymorphic SSRs	% polymorphism	Name of the polymorphic SSR on respective chromosome
1	40	09	22.5	RM495, RM3148, RM7278, RM10167, RM1282, RM10695, RM11069, RM3375, RM220
2	40	10	25.0	RM236, RM279, RM555, RM13129, RM13263, RM13430, RM13433, RM13630, RM5706, RM13996
3	40	09	22.5	RM14239, RM14320, RM14473, RM14603, RM14946, RM15189, RM15441, RM15630, RM16238
4	40	10	25.0	RM335, RM518, RM16356, RM16368, RM16592, RM16626, RM16741, RM17134, RM17263, RM255
5	40	08	20.0	RM122, RM17836, RM17903, RM17941, RM18360, RM18775, RM3476, RM19101
6	40	09	22.5	RM276, RM19711, RM19840, RM20098, RM162, RM20378, RM19422, RM420, RM20773
7	40	06	15.0	RM20913, RM21260, RM320, RM21693, RM21879, RM21024,
8	40	08	20.0	RM22273, RM22659, RM22905, RM23076, RM23356, *xa13prom,* RM23612, RM22914
9	40	08	20.0	RM22431, RM8300, RM219, RM219, RM23959, RM24240, RM242, RM160
10	40	06	15.0	RM24999, RM216, RM25460, RM25557, RM25679, RM25866
11	40	07	17.5	RM26021, RM26550, RM26616, RM287, RM26860, RM26969, *pTA248*
12	40	08	20.0	RM27451, RM27840, RM28130, RM28270, RM28767, RM27789, RM519, RM28766
Total	480	98	20.4	

Supplementary Table 4. Brief summary of foreground and background selection, selection differential, and selection criteria employed in the cross of HUR 917 (RP) and DRR Dhan 42 (donor).

Generation	Selection differential (Δd) for product profile trait					PDI value	AUDPC	Selection criteria
	Name of trait	HUR-917 (RP)	Range before selection	Range in selected top ten plants	Δd			
F_{1s}	-	-	-	-	-	-	-	Hybridity testing with R specific markers
BC_1F_{1s}	DFF (day)	104.0	86.0-112.0	100.0-106.0	1.42	10.22 ± 0.236 to 32.48 ± 0.085	200.54-483.72	FS, BS, Phenome and bioassay
	PH (cm)	110.8	90.82-112.42	104.0- 115.25	1.98			
	L/B ratio	2.892	2.45-3.408	2.82- 3.24	0.09			
	HRR (%)	68.6	52.78- 71.36	58.45-70.25	0.62			
	Aroma	2	0-2	2	0			
BC_2F_{1s}	DFF (day)	106.0	96.0-112.0	100.0- 108.0	1.34	6.05 ± 0.227 to 12.69 ± 0.328	89.82-194.56	FS, BS, Phenome and bioassay
	PH (cm)	106.5	92.85-109.65	102.55-114.05	2.36			
	L/B ratio	2.95	2.52-3.64	2.82 - 3.16	0.06			
	HRR (%)	66.45	52.75-72.65	60.45- 70.82	0.42			
	Aroma	2	0-2	2	0			
BC_3F_1s	DFF (day)	105.0	98.0-109.0	102.0-112.0	2.08	2.48 ± 0.624 to 18.24 ± 0.225	72.24-178.25	FS, BS, Phenome and bioassay analysis
	PH (cm)	112.6	94.45- 110.65	104.55-112.50	1.54			
	L/B ratio	2.91	2.75- 3.46	2.79- 3.085	0.09			
	HRR (%)	66.8	49.64-72.54	59.65 -71.25	1.62			
	Aroma	2	0-2	2	0			
BC_3F_2s	DFF (day)	106	99.0- 112.0	103.0 -110.0	0.09	2.770.87± to 8.89±1.12	79.30 - 101.63	Phenomics analysis
	PH (cm)	109.8	10.35 -110.46	103.0- 112.45	1.28			
	L/B ratio	2.89	2.58- 3.64	2.80-3.21	0.12			
	HRR (%)	62.5	53.0- 73.2	61.5-71.5	0.57			
	Aroma	2	2	2	0			
BC_3F_3s	DFF (day)	112	100.0- 118.0	102.0 – 114.3	0.55	2.34±0.84 to 4.38±1.14	56.35-92.05	BS, Phenome and bioassay analysis
	PH (cm)	110.67	99.0- 118.6	106.0-114.0	1.08			
	L/B ratio	2.90	2.72- 3.64	2.69 - 3.02	0.14			
	HRR (%)	66.74	52.75- 81.62	63.23 - 69.55	0.59			
	Aroma	2	2	2	0			

Note: FS-foreground selection, BS-back ground selection, PDI-plant disease index, AUDPC-area under disease progress curve, Δd-selection differential

Supplementary Table 5. **RP genome recovery in BC_3F_3 generation**

Sl	Near Isogenic line (NILs)	Target gene combination	Markers with RP alleles	Heterozygosity	Donor alleles	% RP genome
1	HR-12-1-4-87-5-2-4	*qDTY*$_{2.2+}$ *qDTY*$_{4.1+}$Xa21+xa13+xa5	93	0	5	93.00
2	HR-12-1-4-87-5-2-8	*qDTY*$_{2.2+}$ *qDTY*$_{4.1+}$Xa21+xa13+xa5	90	3	5	91.53
3	HR-12-1-4-87-5-2-32	*qDTY*$_{2.2+}$ *qDTY*$_{4.1+}$Xa21+xa13+xa5	92	1	5	92.51
4	HR-12-1-4-87-5-2-42	*qDTY*$_{2.2+}$ *qDTY*$_{4.1+}$Xa21+xa13+xa5	91	2	5	92.02
5	HR-12-1-4-87-295-4-3	*qDTY*$_{2.2+}$ *qDTY*$_{4.1+}$Xa21+xa13+xa5	91	2	5	92.02
6	HR-12-1-4-87-295-4-8	*qDTY*$_{2.2+}$ *qDTY*$_{4.1+}$Xa21+xa13+xa5	93	0	5	93.00
7	HR-12-1-4-87-295-4-12	*qDTY*$_{2.2+}$ *qDTY*$_{4.1+}$Xa21+xa13+xa5	92	0	6	92.00
8	HR-12-1-4-87-295-4-19	*qDTY*$_{2.2+}$ *qDTY*$_{4.1+}$Xa21+xa13+xa5	90	3	5	91.53
9	HR-12-70-9-258-145-4-2	*qDTY*$_{2.2+}$ *qDTY*$_{4.1+}$Xa21+xa13+xa5	90	1	7	90.51
10	HR-12-70-9-258-145-4-14	*qDTY*$_{2.2+}$ *qDTY*$_{4.1+}$Xa21+xa13+xa5	92	1	5	92.51
11	HR-12-70-9-258-145-4-23	*qDTY*$_{2.2+}$ *qDTY*$_{4.1+}$Xa21+xa13+xa5	93	0	5	93.00
12	HR-12-70-9-258-169-1-8	*qDTY*$_{2.2+}$ *qDTY*$_{4.1+}$Xa21+xa13+xa5	91	1	6	91.51
13	HR-12-70-9-258-169-1-23	*qDTY*$_{2.2+}$ *qDTY*$_{4.1+}$Xa21+xa13+xa5	91	2	5	92.02
14	HR-12-70-9-258-169-1-38	*qDTY*$_{2.2+}$ *qDTY*$_{4.1+}$Xa21+xa13+xa5	90	3	5	91.53
15	HR-12-70-9-258-223-5-9	*qDTY*$_{2.2+}$ *qDTY*$_{4.1+}$Xa21+xa13+xa5	91	2	5	92.02
16	HR-12-70-9-258-223-5-12	*qDTY*$_{2.2+}$ *qDTY*$_{4.1+}$Xa21+xa13+xa5	90	3	5	91.53
	Average		91.25	1.50	5.25	92.02

Supplementary Table 6. **X^2 analysis for palatability in RP and improved lines based on panel test**

Sample set	No. of panellist	No. of judgments for palatability test in rice		
		Correct	Incorrect	X^2 value
RP+NIL1	25	13	12	1.45
RP+NIL2	25	17	08	1.48
RP+NIL3	25	10	15	1.38
RP+NIL4	25	11	14	1.78
RP+NIL5	25	16	09	1.41
RP+NIL6	25	14	11	1.53
RP+NIL7	25	15	10	1.52
RP+NIL8	25	08	17	0.64
RP+NIL9	25	18	07	1.71
RP+NIL10	25	09	24	0.85
RP+NIL11	25	13	12	1.16
RP+NIL12	25	19	06	1.57
RP+NIL13	25	12	13	1.08
RP+NIL14	25	15	10	1.52
RP+NIL15	25	09	16	1.17
RP+NIL16	25	17	08	1.09

Note: Null hypothesis is rejected as Chi-squire value is not significant, RP line is not significantly differing from NILs for palatability.

Supplementary Table 7a. Duncan's Multiple Range test (DMRT) for analysis of pairwise differences among parents and NILs (BC3F4) of HUR 917/DRR Dhan 42 under non-stress condition (irrigated)

Genotype	DFF	DM	PH	PL	NEBT	SFP	TW	YPP	YPHA	CC	PC	KL	KB	KL AC	ER	AC	HRR	ASV	BLR	GC	CC	VER
HUR-917 (RP)	108.67 abc	138.67 abc	110.33bcd	22.67bc	22.67bc	86.9cdefg	14.63cd	18.25cde	5251cde	60g	23.66ef	4.79^{cde}	1.52^{jk}	7.77^{bc}	1.67^{bc}	23.45^{cd}	66.33^{a}	3^{a}	3.04^{c}	44.00^{b}	60.00^{g}	4.00^{b}
DRR dhan-42 (Donor)	99e	126.67 e	115a	25.67a	25.67a	85.52fgh	25.59a	21.7a	6840a	61g	25de	5.33^{a}	1.91^{a}	6.60^{ef}	1.25^{i}	22.99^{f}	58.33^{h}	3^{a}	2.75^{i}	42.00^{d}	61.00^{g}	4.00^{b}
HR-12-1-4-87-5-2-4	108abc	135.67 c	108.33cdef	23.67b	23.67b	88.14bcde	13.87 d	18.58 bcd	5359bcd	63.67 f	27bc	4.64^{ef}	1.62^{def}	6.11^{g}	1.32^{h}	23.99^{b}	61.33^{g}	3^{a}	2.78^{h}	38.00^{f}	63.67^{f}	4.00^{b}
HR-12-1-4-87-5-2-8	110.33 abc	138.33 abc	105.67ef	21.67bcde	21.67bcde	87.36cdefg	14.49cd	17.77 def	5089def	66e	25de	4.52^{fg}	1.64^{de}	6.69^{e}	1.44^{g}	24.06^{a}	62.33^{f}	3^{a}	2.80^{h}	38.67^{ef}	66.00^{e}	4.00^{b}
HR-12-1-4-87-5-2-32	108.33 abc	136.67 abc	110.33bcd	22bcde	22bcde	88.78abcd	14.82 bcd	17.56 def	5021def	72cd	24e	4.59^{f}	1.68^{bc}	7.90^{ab}	1.67^{ab}	23.86^{bc}	64.33^{bc}	3^{a}	2.81^{h}	39.33^{ef}	72.00^{cd}	4.00^{b}
HR-12-1-4-87-5-2-42	109.67 abc	138abc	107def	22.67bc	22.67bc	83.68h	15.27 bcd	17.37 def	4956.667 def	77.67 ab	23ef	4.65^{def}	1.67^{c}	7.76^{c}	1.68^{ab}	24.16^{a}	64.70^{b}	3^{a}	2.80^{h}	35.00^{g}	77.67^{ab}	4.00^{b}
HR-12-1-4-87-295-4-3	107.33 bcd	136.33 bc	109bcde	22.33bcd	22.33bcd	86.57defg	14.7bcd	18.6bcd	5365.667 bcd	66e	22fg	4.38^{g}	1.54^{ij}	6.70^{e}	1.51^{f}	24.01^{b}	62.64^{ef}	3^{a}	2.83^{g}	40.00^{f}	66.00^{e}	4.25^{a}
HR-12-1-4-87-295-4-8	109.67 abc	140ab	109bcde	20.33de	20.33de	85.27gh	16.31 b	17.47 def	4989.667 def	71d	28ab	4.38^{g}	1.57^{h}	6.50^{f}	1.47^{g}	23.30^{f}	63.15^{f}	3^{a}	2.80^{h}	39.00^{ef}	71.00^{d}	4.25^{a}
HR-12-1-4-87-295-4-12	109.67 abc	139.33 abc	109bcde	20e	20e	86.35efg	16.05 bc	18.69 bcd	5395.667 bcd	67.67 e	25de	4.62^{f}	1.62^{def}	7.50^{d}	1.60^{e}	23.97^{b}	63.40^{cde}	3^{a}	2.85^{g}	39.33^{ef}	67.67^{e}	4.00^{b}
HR-12-1-4-87-295-4-19	110.33 abc	137.67 abc	107.67def	22bcde	22bcde	89.04abc	15.22 bcd	19.36 bc	5620.333 bc	79a	21gh	4.59^{f}	1.60^{fg}	7.57^{d}	1.64^{bcde}	23.09^{f}	62.29^{f}	3^{a}	2.89^{f}	45.00^{a}	79.00^{a}	4.00^{b}
HR-12-70-9-258-145-4-2	103.33 d	131.33 d	109bcde	22.33bcd	22.33bcd	87.72cdef	14.5cd	16.63f	4711f	76.33 b	29ab	4.90^{bc}	1.70^{b}	7.87^{abc}	1.63^{cde}	23.49^{bcd}	64.67^{b}	3^{a}	2.91^{f}	40.00^{f}	76.33^{b}	4.00^{b}
HR-12-70-9-258-145-4-14	106.67 cd	135.67 c	101.67g	22bcde	22bcde	87.62cdef	15.3bcd	18.58 bcd	5360bcd	70.67 d	20h	4.81^{cd}	1.59^{g}	7.87^{abc}	1.65^{bcd}	22.97^{f}	64.17^{bc}	3^{a}	3.03^{c}	42.00^{d}	70.67^{d}	4.00^{b}
HR-12-70-9-258-145-4-23	112.67 a	140.67 a	111.67abc	22.5bc	22.5bc	86.67defg	15.08 bcd	17.47 def	4991def	73.67 c	24e	5.03^{b}	1.62^{defg}	7.90^{ab}	1.64^{bcde}	22.81^{e}	63.95^{bcd}	3^{a}	3.12^{a}	44.00^{b}	73.67^{c}	4.00^{b}
HR-12-70-9-258-169-1-8	109.67 abc	138.33 abc	105fg	20.67cde	20.67cde	87.06cdefg	15.26 bcd	19.69 b	5730b	60g	26cd	4.88^{bc}	1.54^{i}	7.87^{abc}	1.63^{cde}	22.77^{e}	64.62^{b}	3^{a}	3.02^{e}	41.00^{e}	60.00^{g}	4.00^{b}
HR-12-70-9-258-169-1-23	107.33 bcd	136bc	109.33bcde	23.33b	23.33b	90.74a	14.6cd	18.26cde	5252.333 cde	62fg	29ab	4.92^{bc}	1.64^{d}	7.90^{ab}	1.62^{fg}	23.08^{f}	64.67^{b}	3^{a}	3.03^{d}	40.00^{f}	62.00^{fg}	4.00^{b}
HR-12-70-9-258-169-1-38	103de	132d	106ef	22.67bc	22.67bc	88.88abcd	14.01 d	17.56 def	5020def	60.33 g	29ab	4.89^{bc}	1.60^{fg}	7.93^{a}	1.60^{e}	22.88^{e}	64.52^{b}	3^{a}	3.06^{b}	41.00^{e}	60.33^{g}	4.00^{b}
HR-12-70-9-258-223-5-9	112ab	139.67 abc	110.33bcd	23.67b	23.67b	87.68cdef	14.55cd	17.94 de	5147def	60g	30a	4.93^{bc}	1.61^{efg}	7.50^{d}	1.52^{f}	22.97^{f}	62.86^{ef}	3^{a}	3.05^{b}	40.00^{f}	60.00^{g}	4.00^{b}
HR-12-70-9-258-223-5-12	110.33 abc	138.33 abc	112.33ab	23.33b	23.33b	90.28ab	14.6cd	18.1cde	4867ef	61.67 fg	29ab	4.66^{def}	1.51^{k}	7.90^{ab}	1.71^{a}	22.23^{f}	64.84^{b}	3^{a}	3.07^{b}	42.67^{c}	61.67^{fg}	4.00^{b}

Supplementary Table 7b. Duncan's Multiple Range test (DMRT) for analysis of pairwise differences among parents and NILs (BC3F4) of HUR 917/DRR Dhan 42 under stress condition (drought)

Genotype	DFF	DM	PH	PL	EBT	SFP	YPP	YPHA	TW	LR	CC	PC	KL	KB	HRR	GC	ER	CC	KLBR	KLAC	ac	VER	ASV
HUR-917 (RP)	115.67ab	142.33a	107.33a	23.33a	8d	57.19b	6.6e	2200e	12.41gh	7a	22g	27.67j	4.88^{e}	1.51^{h}	63.59bcde	41.00cdef	4a	22.00^{g}	3.23^{cdefg}	7.63^{f}	23.09^{abc}	1.56^{h}	3^{a}
DRR dhan-42 (Donor)	101.33e	126.33g	100ab	22.33ab	10.33abc	74.61a	9.23abcd	3078abcd	24.03a	5b	37.67cde	71.67i	5.25^{a}	1.90^{a}	62.21f	40.67cdef	4a	37.67^{cde}	2.76^{h}	8.03^{ab}	22.89^{abcd}	1.53^{r}	3^{a}
HR-12-1-4-87-5-2-4	115abc	139abcde	99.67ab	22ab	11ab	70.56a	8.6bcd	2866.33bcd	14bc	5b	38.33c	80f	5.08^{bc}	1.56^{cde}	64.23abcd	41.67bcde	4a	38.33^{c}	3.25^{cdef}	7.83^{cde}	22.82^{bcd}	1.54^{o}	3^{a}
HR-12-1-4-87-5-2-8	115abc	140abcd	98.33bc	22ab	10.67ab	69.46a	7.87de	2622de	14.31bc	4.33b	36.33ef	81.67e	5.02^{bc}	1.59^{bc}	64.02abcde	42.67bc	4a	36.33^{ef}	3.15^{g}	7.82^{cde}	22.78^{cd}	1.55^{j}	3^{a}
HR-12-1-4-87-5-2-32	112.67bcd	137.67bcdef	97.33bc	21.67abc	10abc	75.32a	8.6bcd	2866.67bcd	12.51g	4.33b	42.3a	84d	4.91^{de}	1.55^{defg}	64.41ab	41.00cdef	4a	42.31^{a}	3.17^{fg}	7.70^{ef}	23.49^{a}	1.56^{g}	3^{a}
HR-12-1-4-87-5-2-42	113.67abc	139.33abcde	97bc	21.67abc	11ab	70.73a	8.5bcd	2833.33bcd	13cde	3c	36f	90c	5.09^{b}	1.52^{fgh}	63.23e	41.00cdef	4a	36.00^{f}	3.34^{ab}	7.93^{bcd}	23.46^{ab}	1.55^{k}	3^{a}
HR-12-1-4-87-295-4-3	112.33bcd	137.67bcdef	96.67bc	21.67abc	9.67abcd	73.003a	8.37bcd	2789bcd	12.73defg	3c	40b	92b	5.09^{b}	1.56^{def}	64.10abcde	43.00ab	4a	40.00^{b}	3.27^{bcde}	7.90^{bcd}	23.00^{abc}	1.55^{l}	3^{a}
HR-12-1-4-87-295-4-8	112.33bcd	138.33bcdef	96bc	21.33bc	9.33bcd	73.63a	7.77de	2589de	12.07h	3c	38cd	94a	5.09^{b}	1.60^{b}	63.83abcde	44.67a	4a	38.00^{cd}	3.18^{defg}	7.81^{de}	23.13^{abc}	1.53q	3^{a}
HR-12-1-4-87-295-4-12	112.33bcd	137.33cdef	95.67bc	21.33bc	8.67cd	78.11a	9.5abc	3166.67abc	13.09bcd	3c	38cd	77.07g	4.92^{de}	1.55^{defg}	63.47cde	41.67bcde	4a	38.00^{cd}	3.18^{efg}	7.80^{de}	23.15^{abc}	1.58^{f}	3^{a}
HR-12-1-4-87-295-4-19	114abc	138bcdef	95bc	21bcd	14.67cd	74.46a	9.6ab	3200ab	13.34bc	3c	40.33b	80.2f	4.87^{e}	1.52^{fgh}	63.78abcd	39.00f	4a	40.33^{b}	3.20^{def}	7.83^{cde}	22.33^{d}	1.61	3^{a}

															e				^{g}			^{a}	
HR-12-70-9-258-145-4-2	108.67d	136ef	95bc	21bcd	14.33cd	74.25a	8.73abcd	2911abcd	13.92bc	3c	42a	72i	4.99^{cd}	1.52^{fgh}	63.39de	41.00cdef	4a	42.00^{a}	3.28^{bcd}	7.93^{bcd}	22.28^{d}	1.59^{d}	3^{a}
HR-12-70-9-258-145-4-14	109d	134.67f	94.67bc	21bcd	8.67cd	77.92a	10.17a	3389a	12.57fg	3c	37.33cdef	74.1h	5.06^{bc}	1.54^{efgh}	64.13abcd	40.67cdef	4a	37.33^{cdef}	3.28^{bcd}	7.90^{bcd}	22.85^{abcd}	1.56^{i}	3^{a}
HR-12-70-9-258-145-4-23	116ab	141abc	94.67bc	20.67bcd	11.33a	76.56a	9.8ab	3266.67ab	13.02cde	3c	40b	78.13g	5.03^{bc}	1.58^{bcd}	63.48cde	42.00bcd	4a	40.00^{b}	3.18^{defg}	7.80^{de}	23.13^{abc}	1.55^{m}	3^{a}
HR-12-70-9-258-169-1-8	113.67abc	139.33abcde	94bc	20.66bcd	9.67abcd	74.59a	9.6ab	3200ab	13cde	3c	38.84bc	82.73de	4.91^{de}	1.54^{efgh}	64.55a	40.00def	4a	38.84^{bc}	3.19^{defg}	7.80^{de}	23.17^{abc}	1.59^{e}	3^{a}
HR-12-70-9-258-169-1-23	111cd	136.33def	93.67bc	20.67bcd	10.33abc	72.44a	10.15a	3382.33a	13.4b	3c	37.67cde	80.2f	4.92^{de}	1.51^{h}	63.50cde	41.00cdef	4a	37.67^{cde}	3.25^{bcdef}	7.83^{cde}	23.26^{abc}	1.59^{c}	3^{a}
HR-12-70-9-258-169-1-38	109.33d	134.67f	93bc	20.33bcd	9.33bcd	69.17a	8cde	2666.67cde	12.91def	3c	36.67def	81.95e	5.04^{bc}	1.52^{gh}	64.12abcde	39.33f	4a	36.67^{ef}	3.32^{bc}	7.80^{de}	23.22^{abc}	1.54^{n}	3^{a}
HR-12-70-9-258-223-5-9	116ab	141.33ab	90.33bc	19.67cd	10abc	72.73a	8.97abcd	2988.67abcd	12.4gh	3c	40b	82.07e	5.20^{a}	1.52^{gh}	64.33abc	40.00def	4a	40.00^{b}	3.42^{a}	7.98^{abc}	22.97^{abc}	1.53^{p}	3^{a}
HR-12-70-9-258-223-5-12	114.33abc	139abcde	89.33c	19.33d	10.67ab	72.82a	9.1abcd	3033.33abcd	14.65efg	2.33c	40.33b	82.59e	5.08^{bc}	1.60^{b}	63.44cde	39.67ef	4a	40.33^{b}	3.17^{fg}	8.11^{a}	23.14^{abc}	1.60^{b}	3^{a}

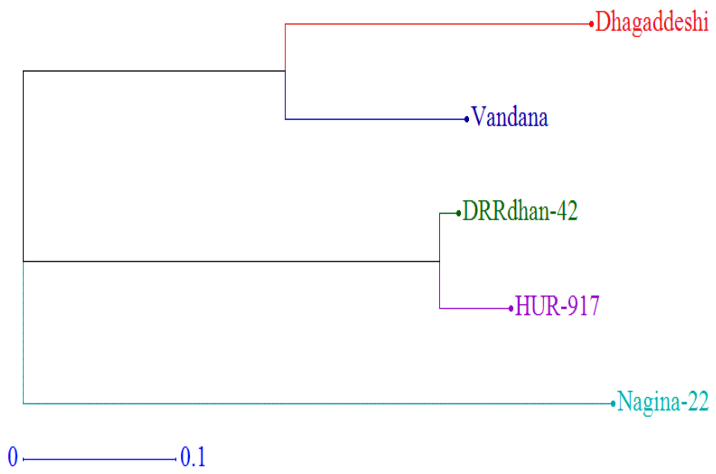

Supplementary Figure 1. An unweighted pair-group method with arithmetic means (UPGMA) dendrogram for 5 rice genotypes based on genetic similarity by 41 simple sequence repeat markers.

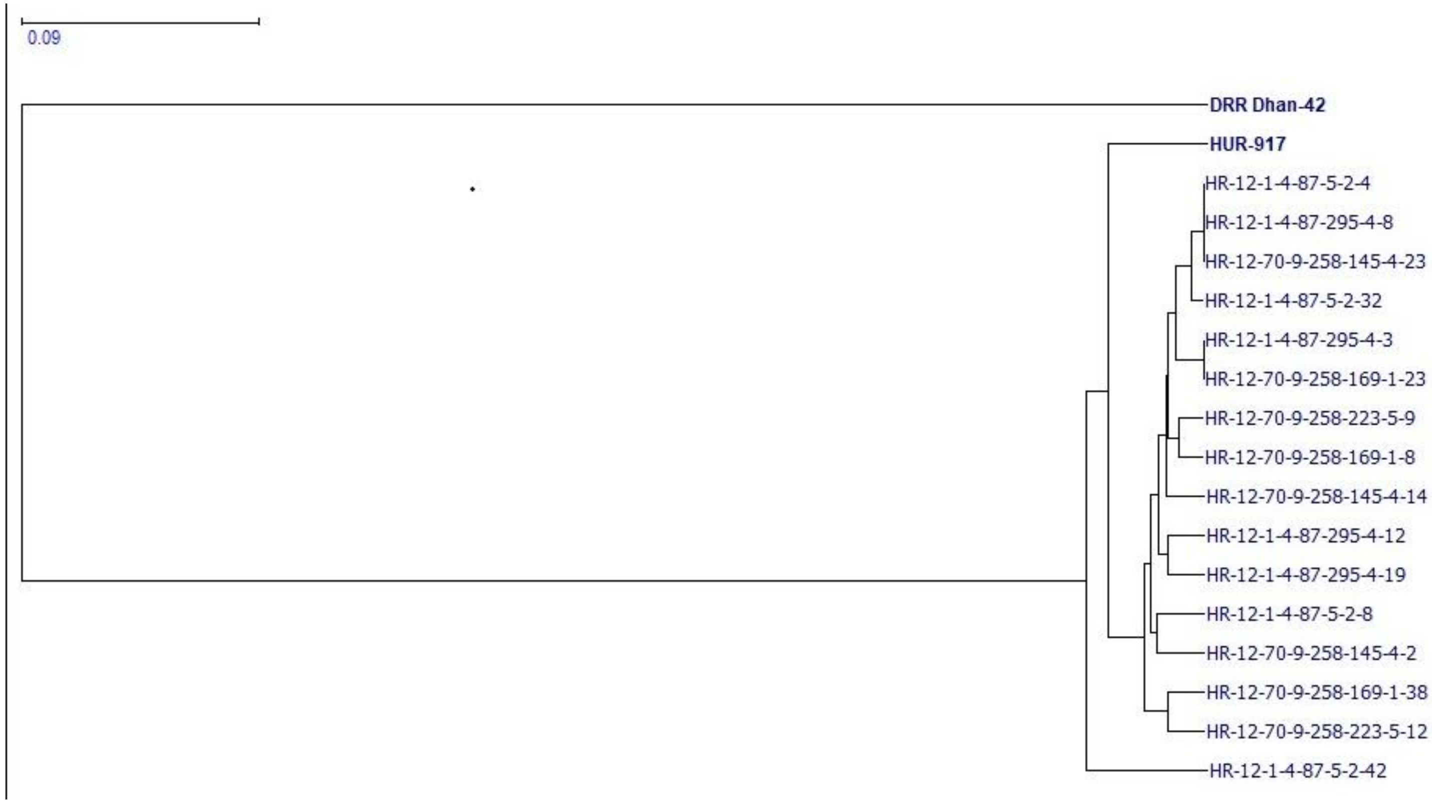

Supplementary **Figure 2.** An unweighted pair-group method with arithmetic means (UPGMA) dendrogram for 18 rice genotypes (parents +NILs) based on genetic similarity by 98 simple sequence repeat markers.

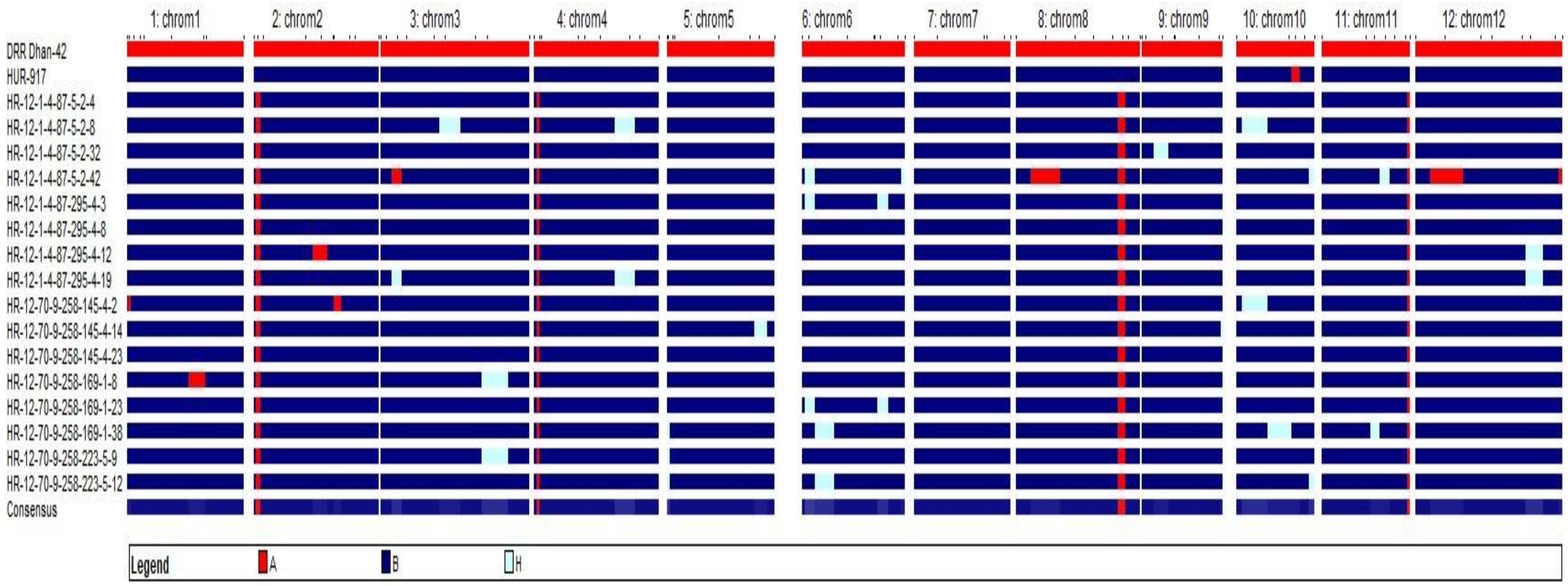

Supplementary Figure 3. Extant of RP genome recovery in derivative NILs (1-16 no.)

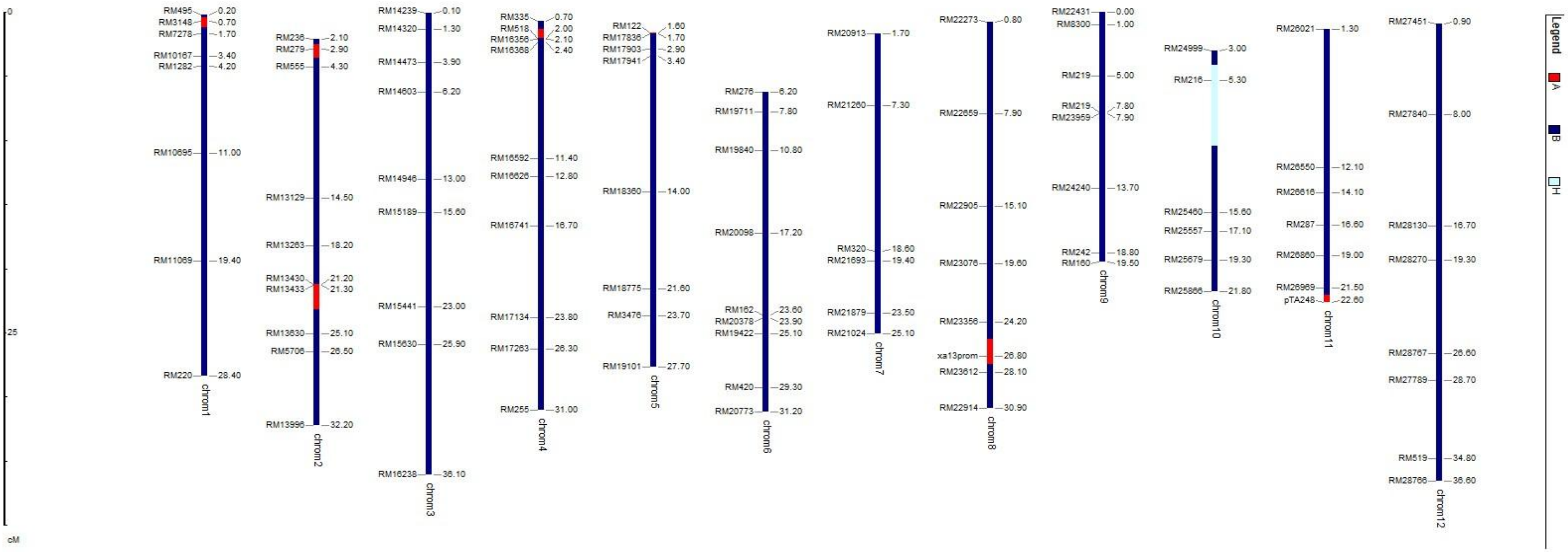

Ind no:11 [HR-12-70-9-258-145-4-2] - Chrom-12

Supplementry Figure 4. Physical position of SSR markers used for gene introgression and genome recovery of the recurrent parent

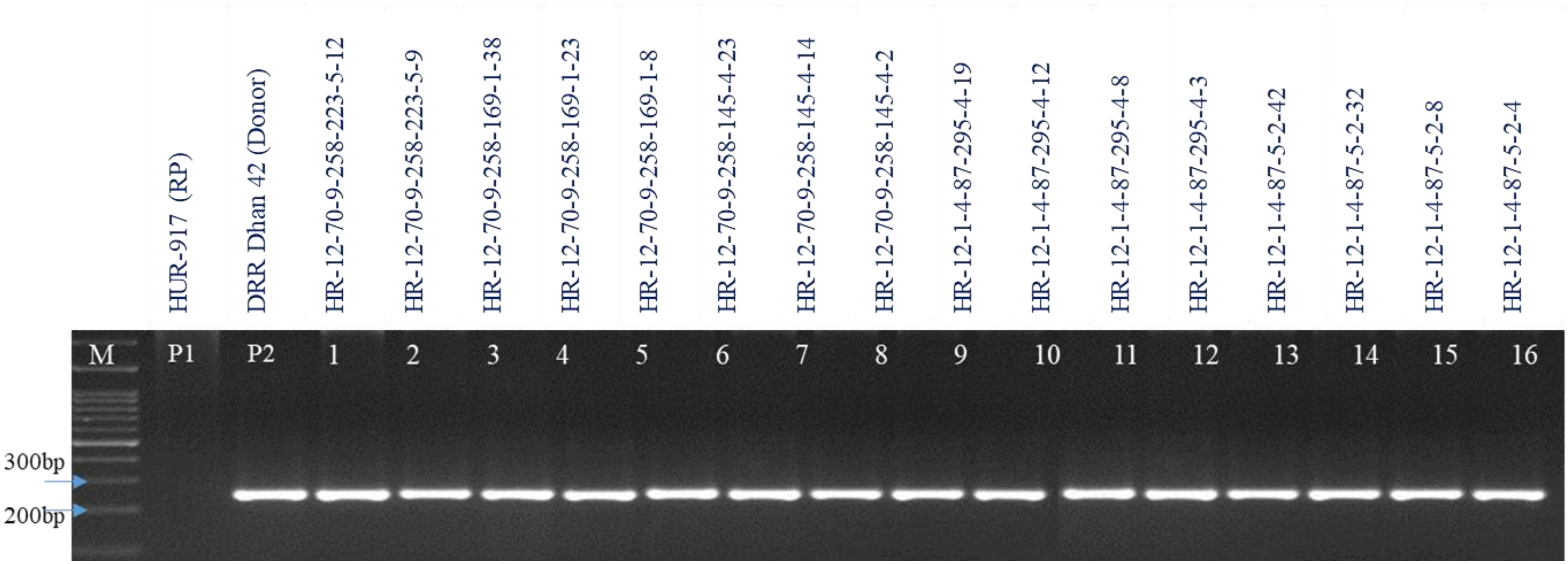

Supplementary Figure 5a. PCR amplification of BADH2 gene in BC3F4 NILs using FMbadh2-E7 functional marker; M, Marker; P1- DR Dhan 42 (shows no band), P2-RP (Imp. HUR 917) and lane 3-16 are NILs.

Chromosome 1

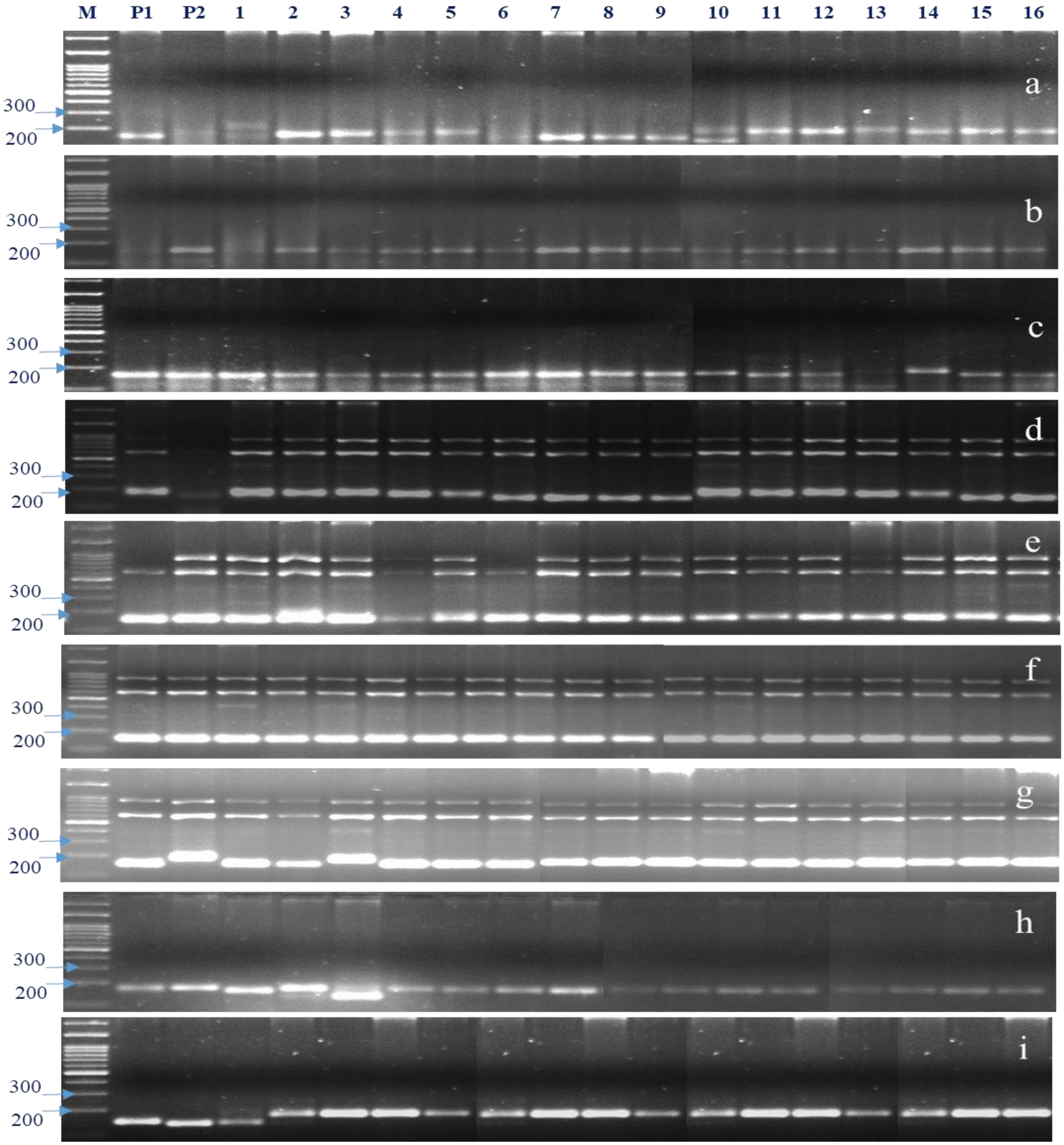

Supplementary figure 5b: Back-ground genome recovery analysis through in in BC₃F₃ generation (a) RM495, (b) RM3148, (c) RM7278, (d) RM10167, (e) RM1282, (f) RM10695, (g) RM11069, (h) RM3375, (i) RM220

Chromosome 2

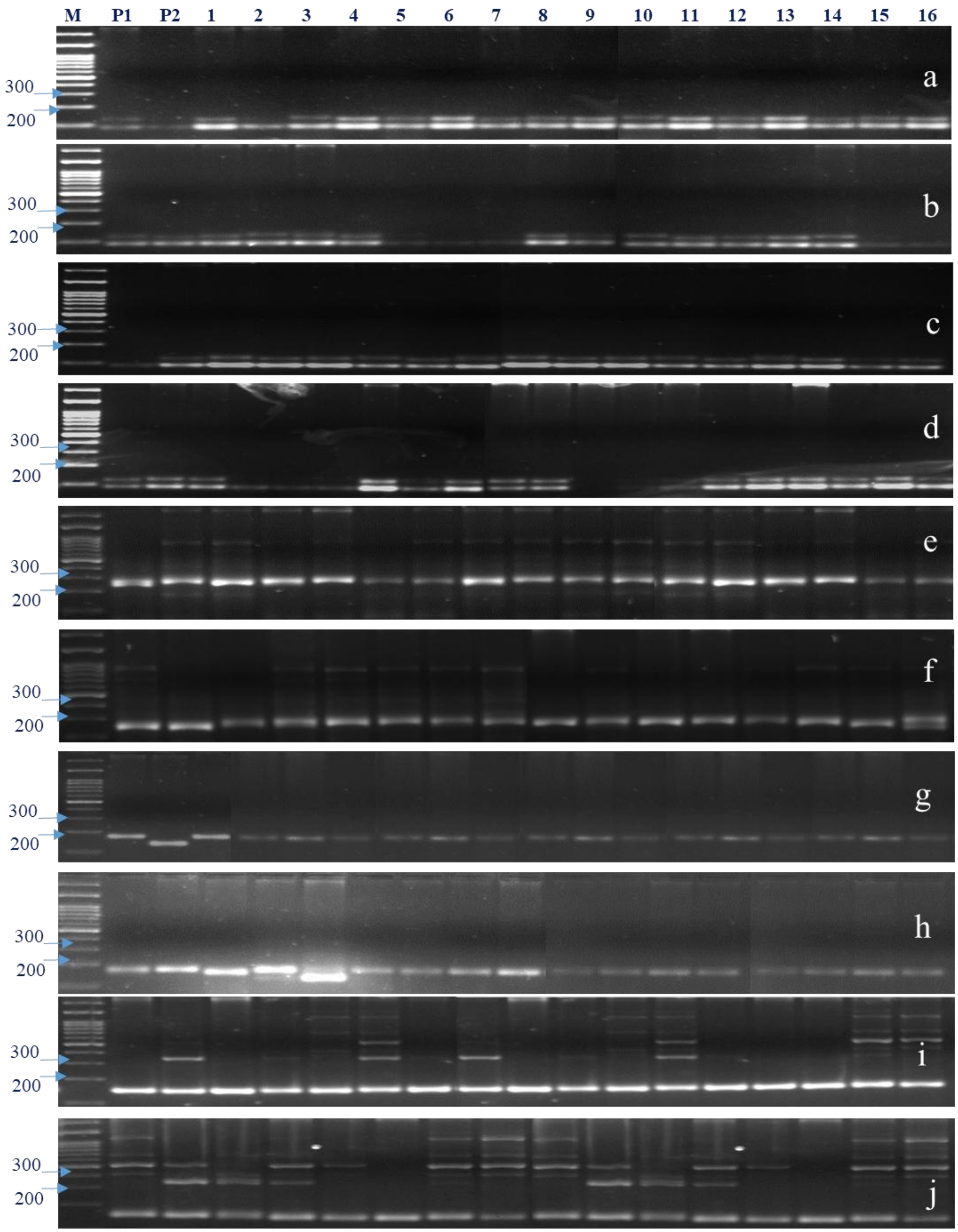

Supplementary figure 5c: Back-ground genome recovery analysis through in in BC_3F_3 generation (a) RM236, (b) RM279, (c) RM555, (d) RM13129, (e) RM13430, (f) RM13433, (g) RM13630, (h) RM5706, (i) RM3996, (j) RM13263

Chromosome 3

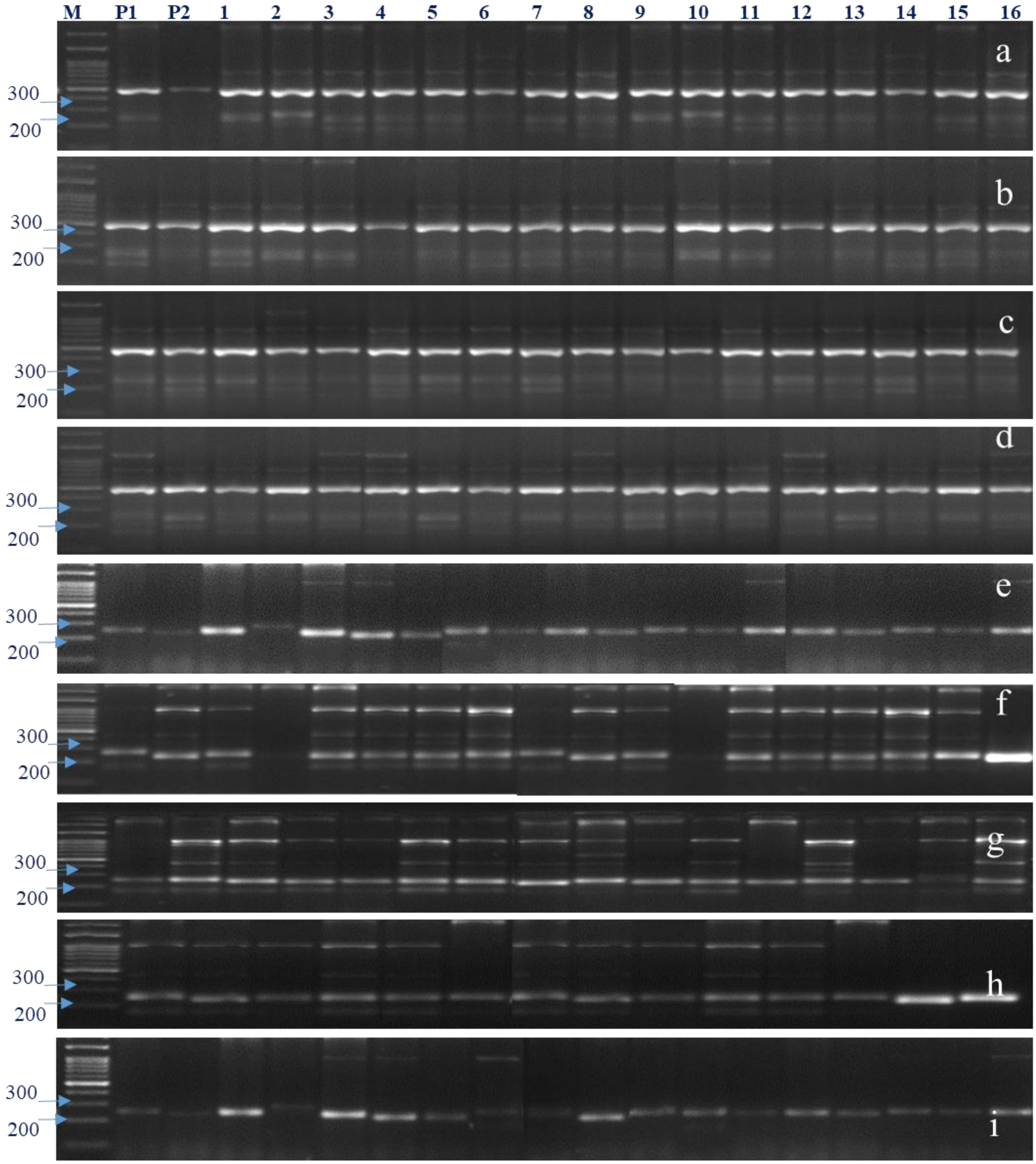

Supplementary figure 5d: Back-ground genome recovery analysis through in in BC$_3$F$_3$ generation (a) RM14239, (b) RM14320, (c) RM14473, (d) RM14603, (e) RM14946, (f) RM15189, (g) RM15441, (h) RM15630, (i) RM16238

Chromosome 4

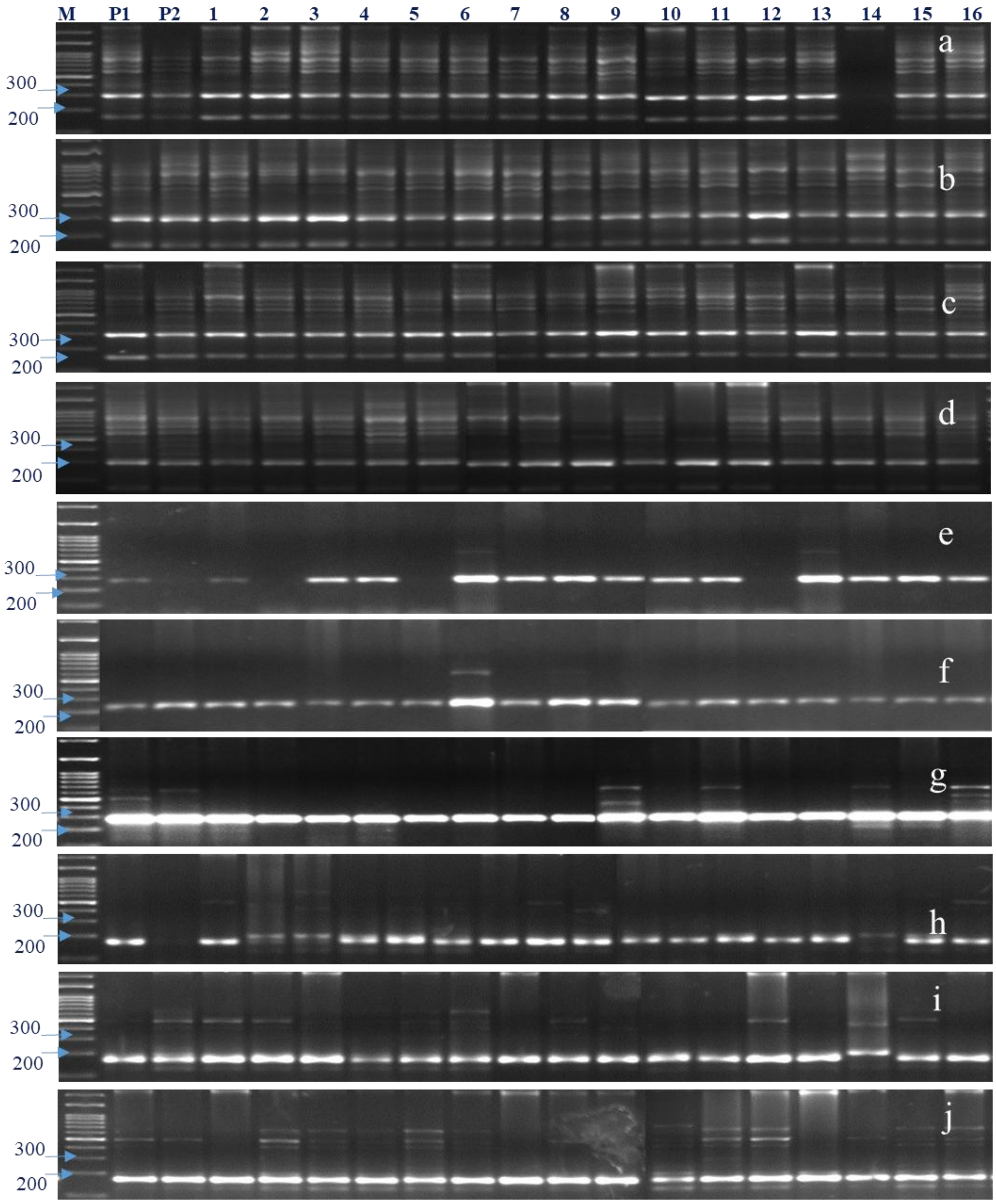

Supplementary figure 5e: Back-ground genome recovery analysis through in in BC_3F_3 generation (a) RM335, (b) RM518, (c) RM16356, (d) RM16368, (e) RM16592, (f) RM16626, (g) RM16741, (h) RM17134, (i) RM17263, (j) RM255

Chromosome 5

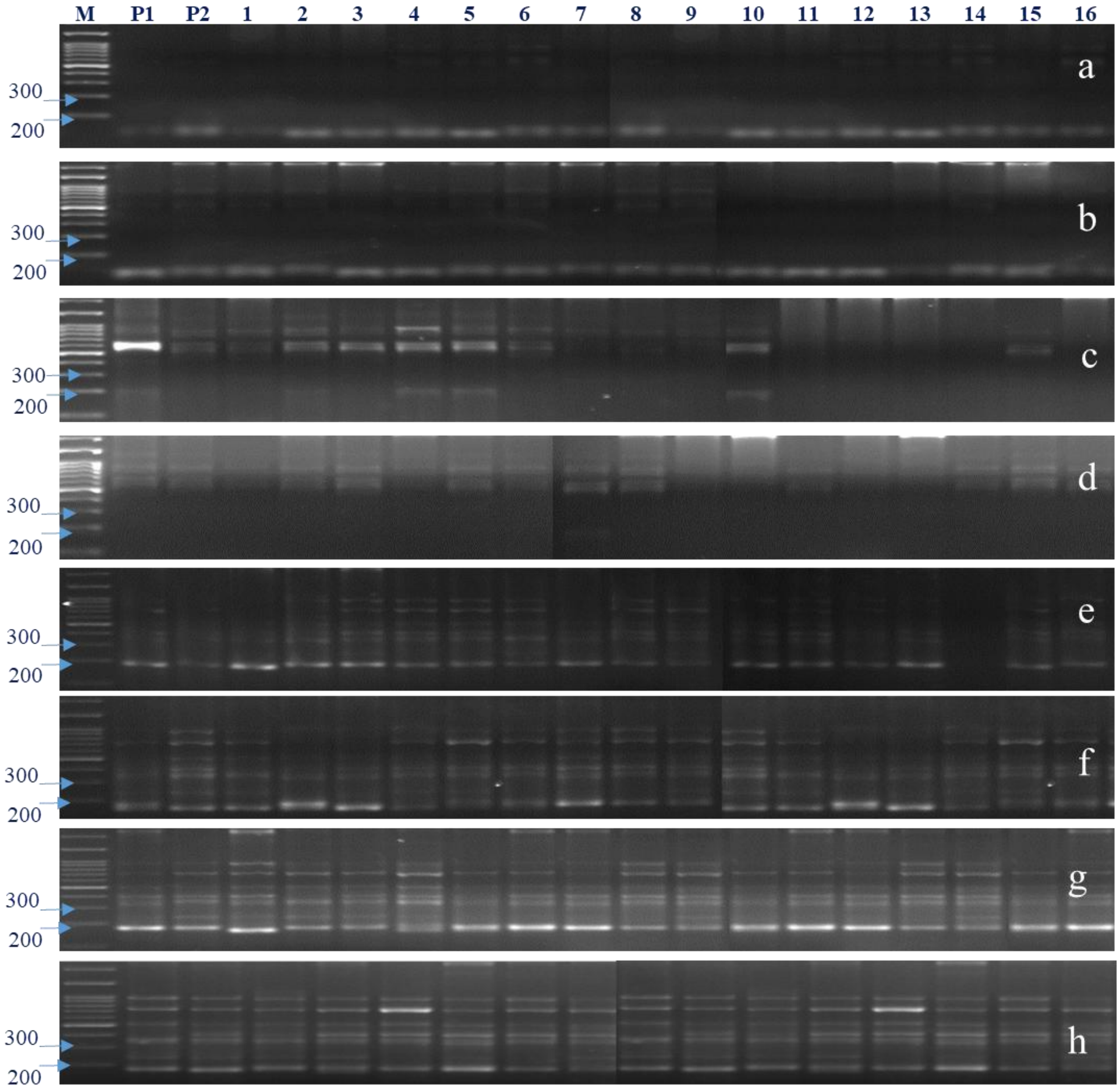

Supplementary figure 5f: Back-ground genome recovery analysis through in in BC_3F_3 generation (a) RM122, (b) RM17836, (c) RM17903, (d) RM17941, (e) RM18360, (f) RM18775, (g) RM3476, (h) RM19101

Chromosome 6

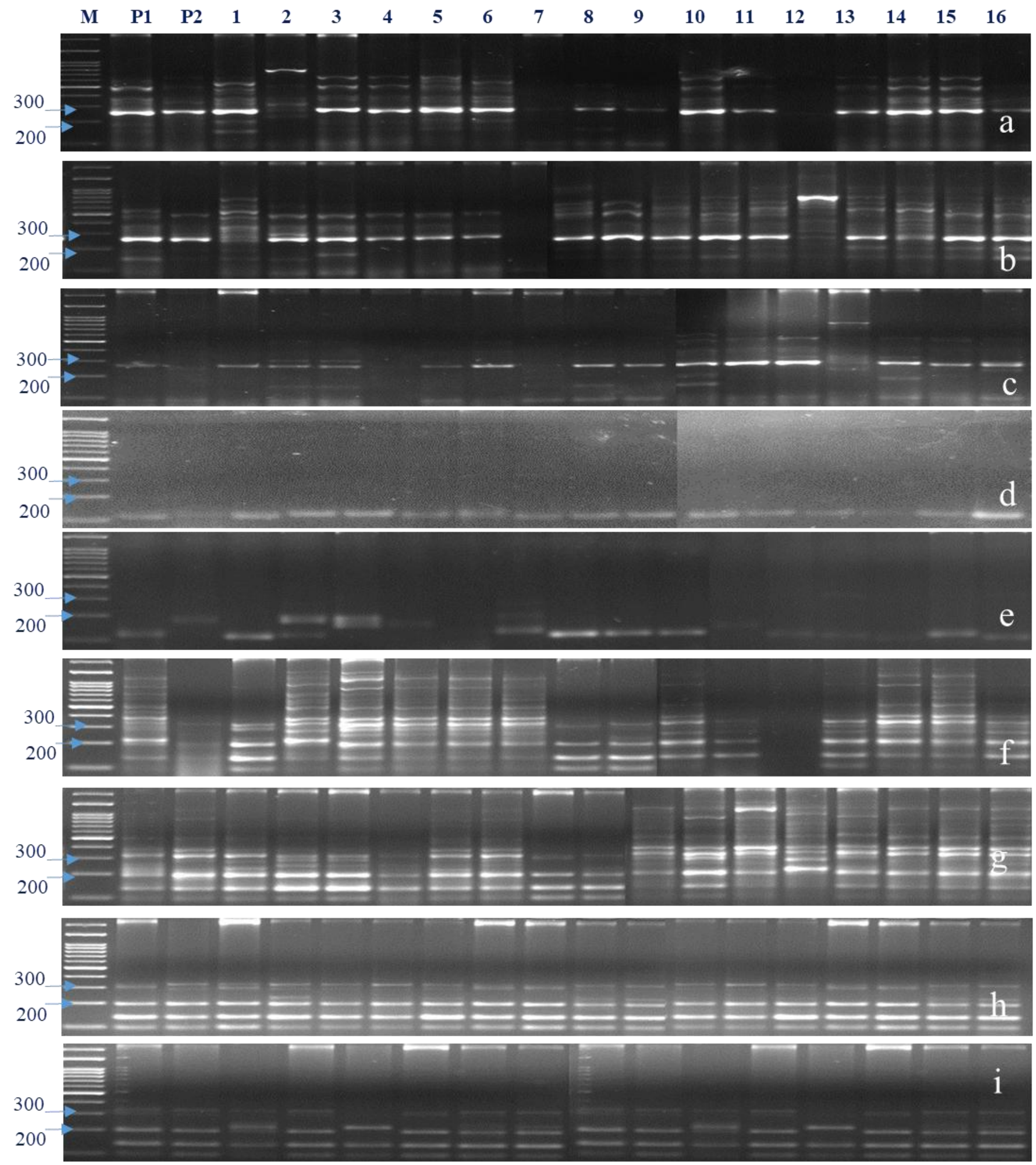

Supplementary figure 5g: Back-ground genome recovery analysis through in in BC_3F_3 generation (a) RM276, (b) RM19711, (c) RM19840, (d) RM20098, (e) RM162, (f) RM20378, (g) RM19422, (h) RM420, (i) RM20773

Chromosome 7

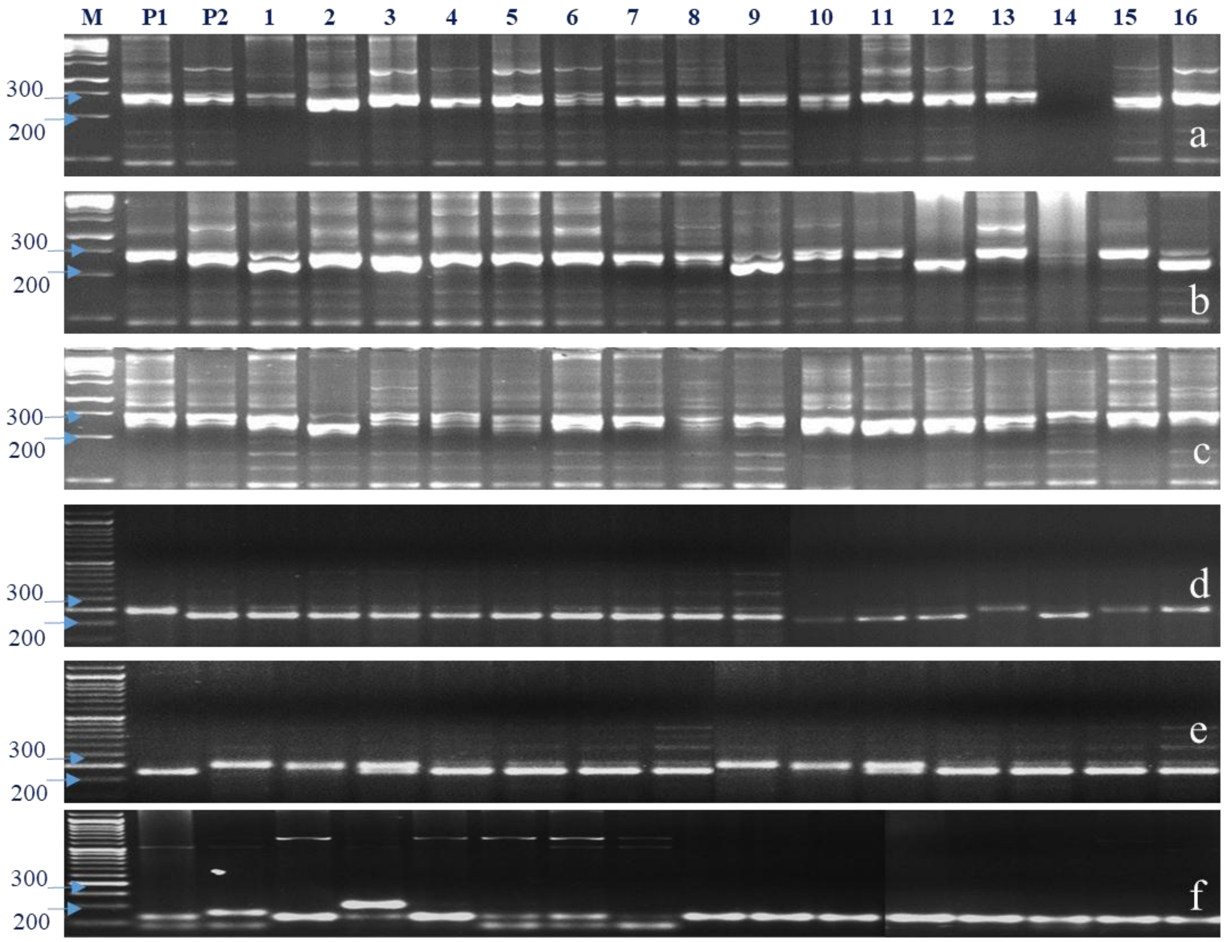

Supplementary figure 5h: Back-ground genome recovery analysis through in in BC_3F_3 generation (a) RM20913, (b) RM21260, (c) RM320, (d) RM21693, (e) RM21879, (f) RM21024

Chromosome 8

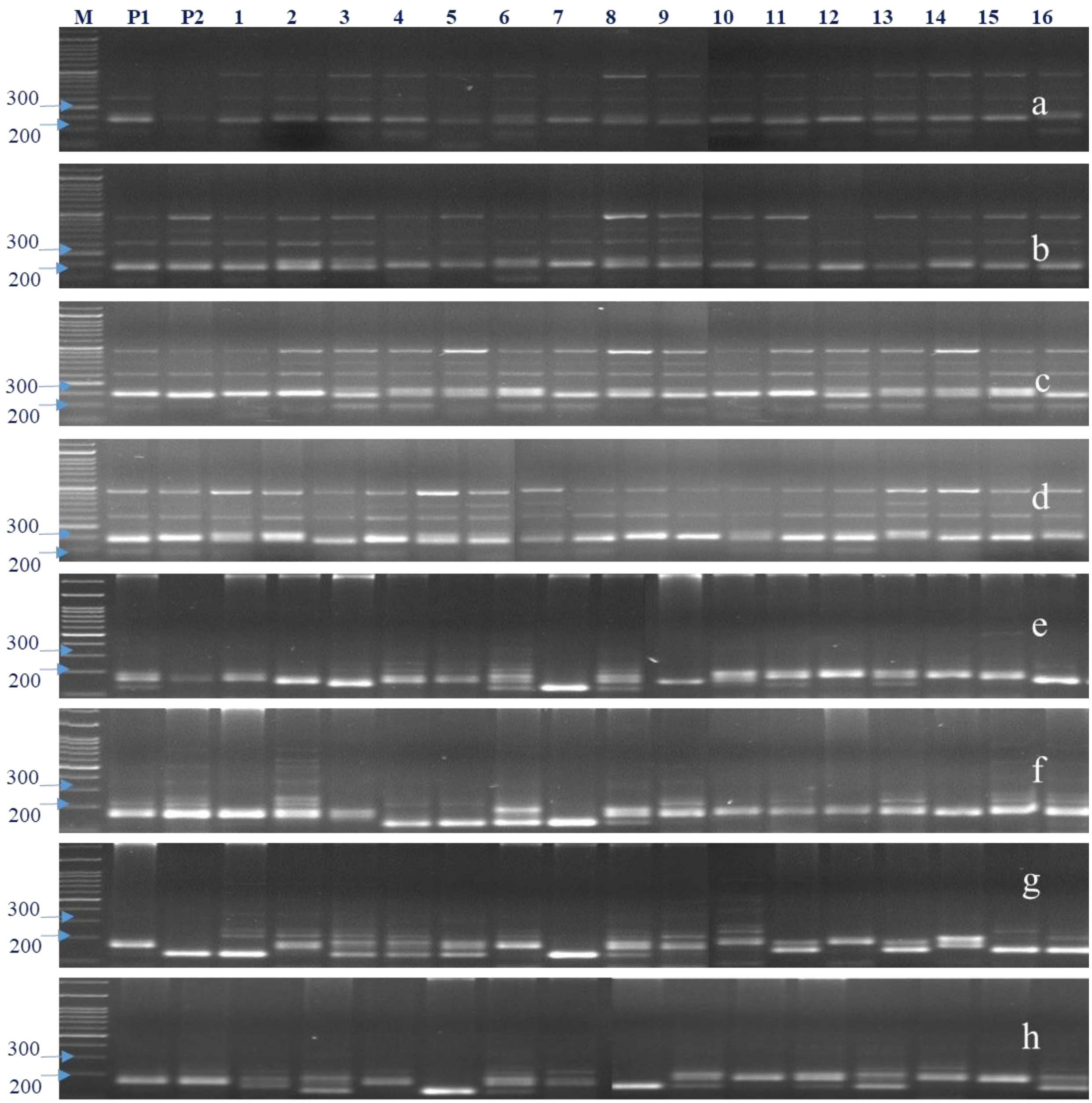

Supplementary figure 5i: Back-ground genome recovery analysis through in in BC_3F_3 generation (a) RM22273, (b) RM22659, (c) RM22905, (d) RM23076, (e) RM23356, (f) RM23612, (g) RM22914

Chromosome 9

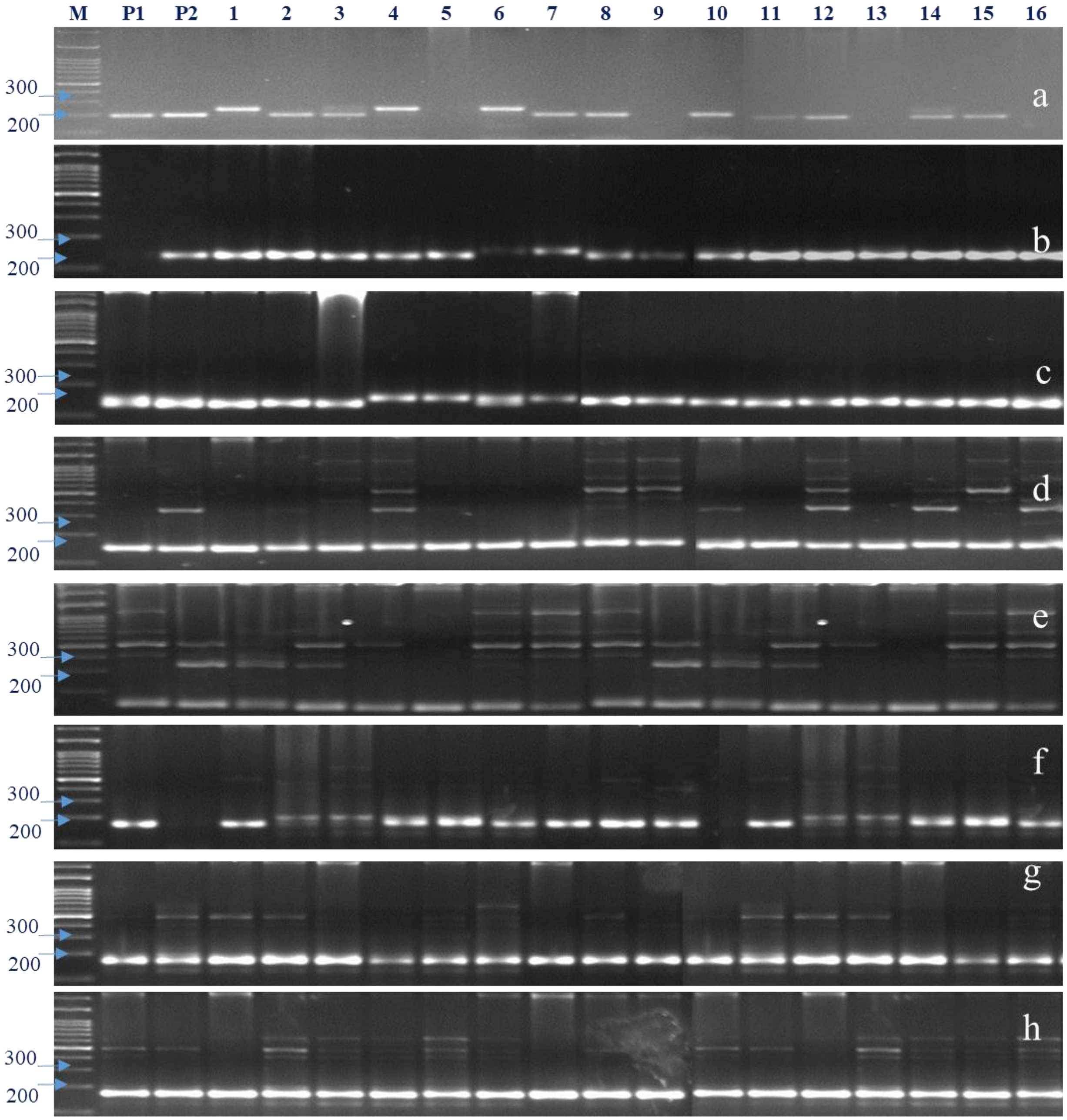

Supplementary figure 5j: Back-ground genome recovery analysis through in in BC$_3$F$_3$ generation (a) RM22431, (b) RM8300, (c) RM219, (d) RM23959, (e) RM24240, (f) RM242, (g) RM160

Chromosome 10

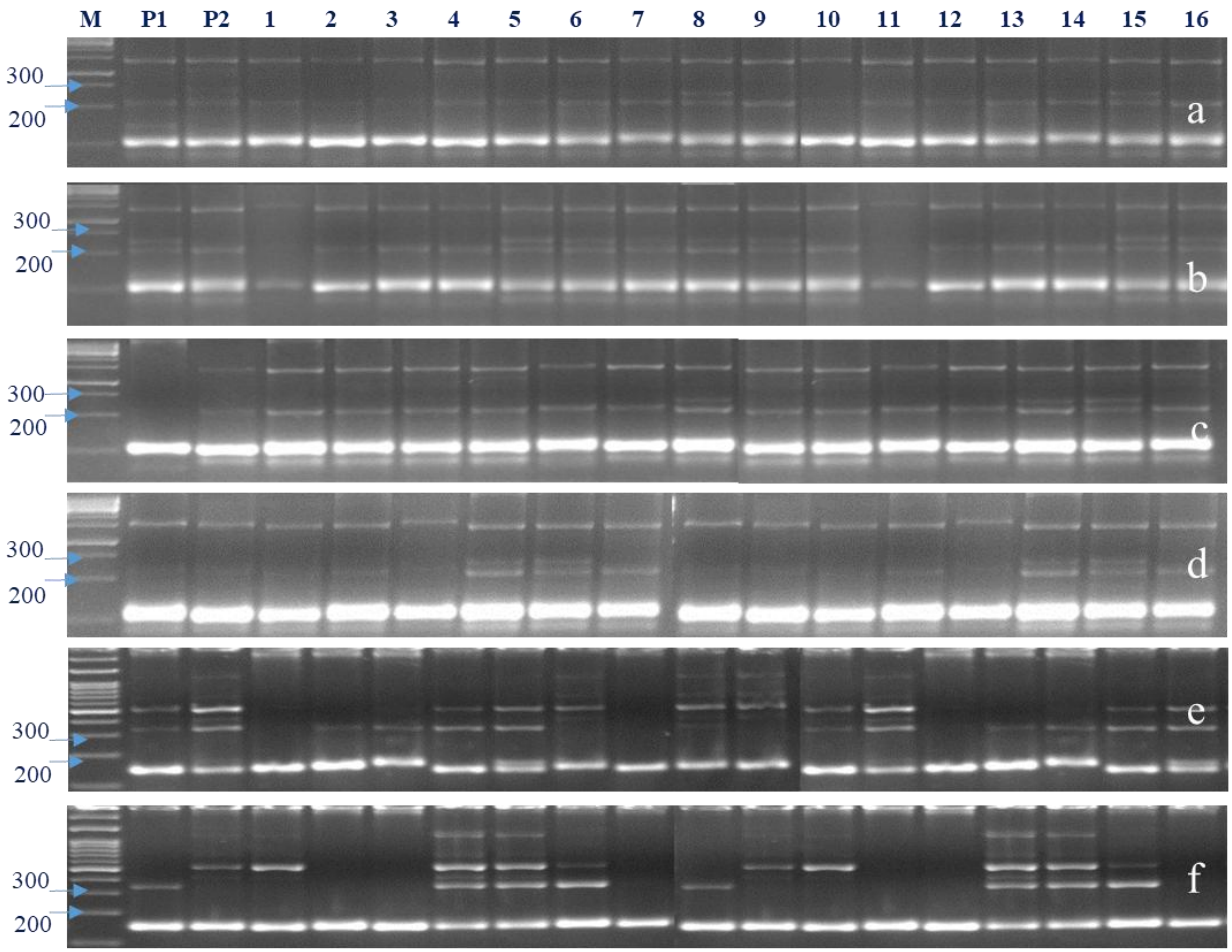

Supplementary figure 5k: Back-ground genome recovery analysis through in in BC_3F_3 generation (a) RM2499, (b) RM216, (c) RM25460, (d) RM2557, (e) RM25679, (f) RM25866

Chromosome 11

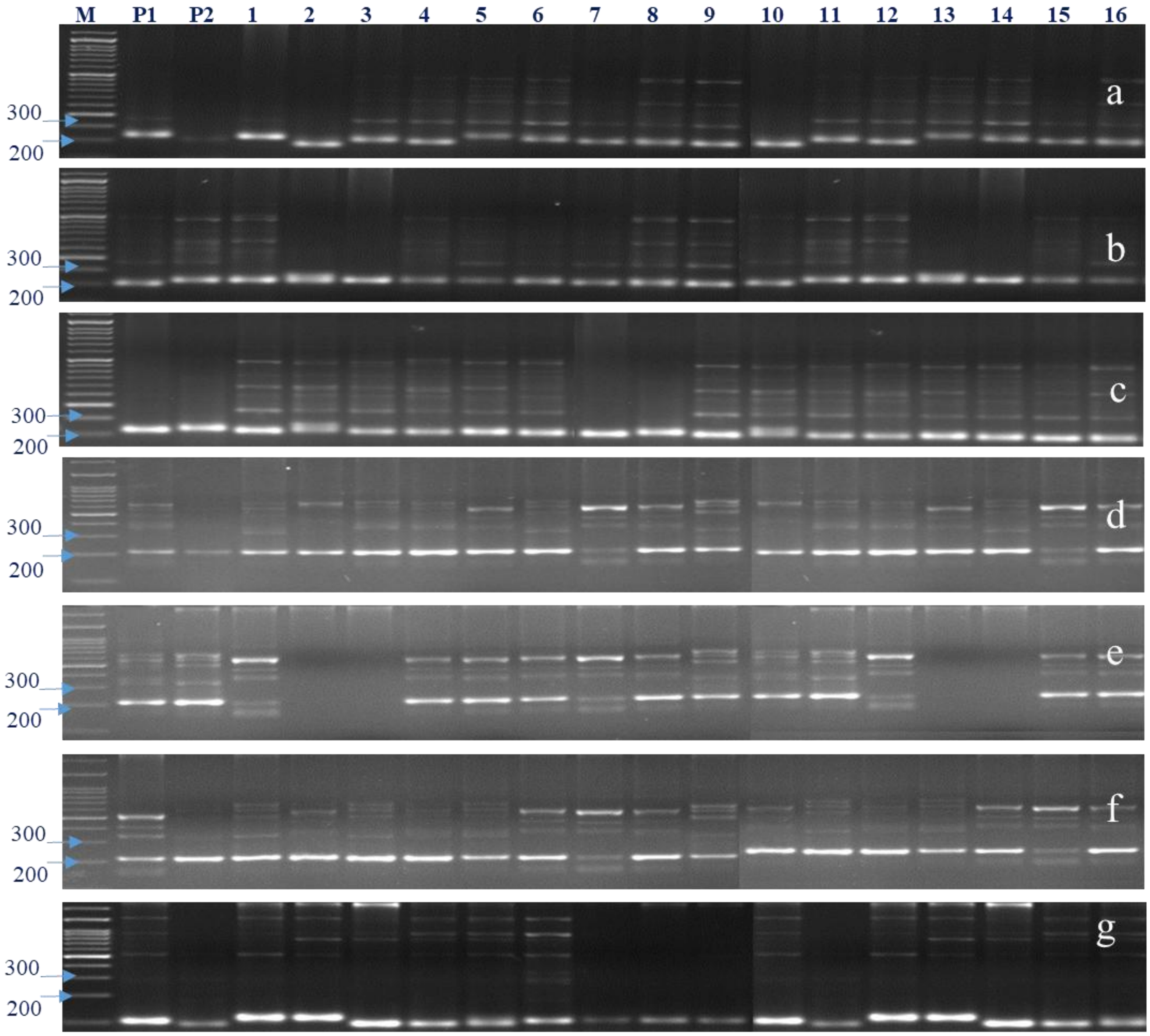

Supplementary figure 5l: Back-ground genome recovery analysis through in in BC_3F_3 generation (a) RM26021, (b) RM26550, (c) RM26616, (d) RM287, (e) RM162, (f) RM26860, (g) RM26959

Chromosome 12

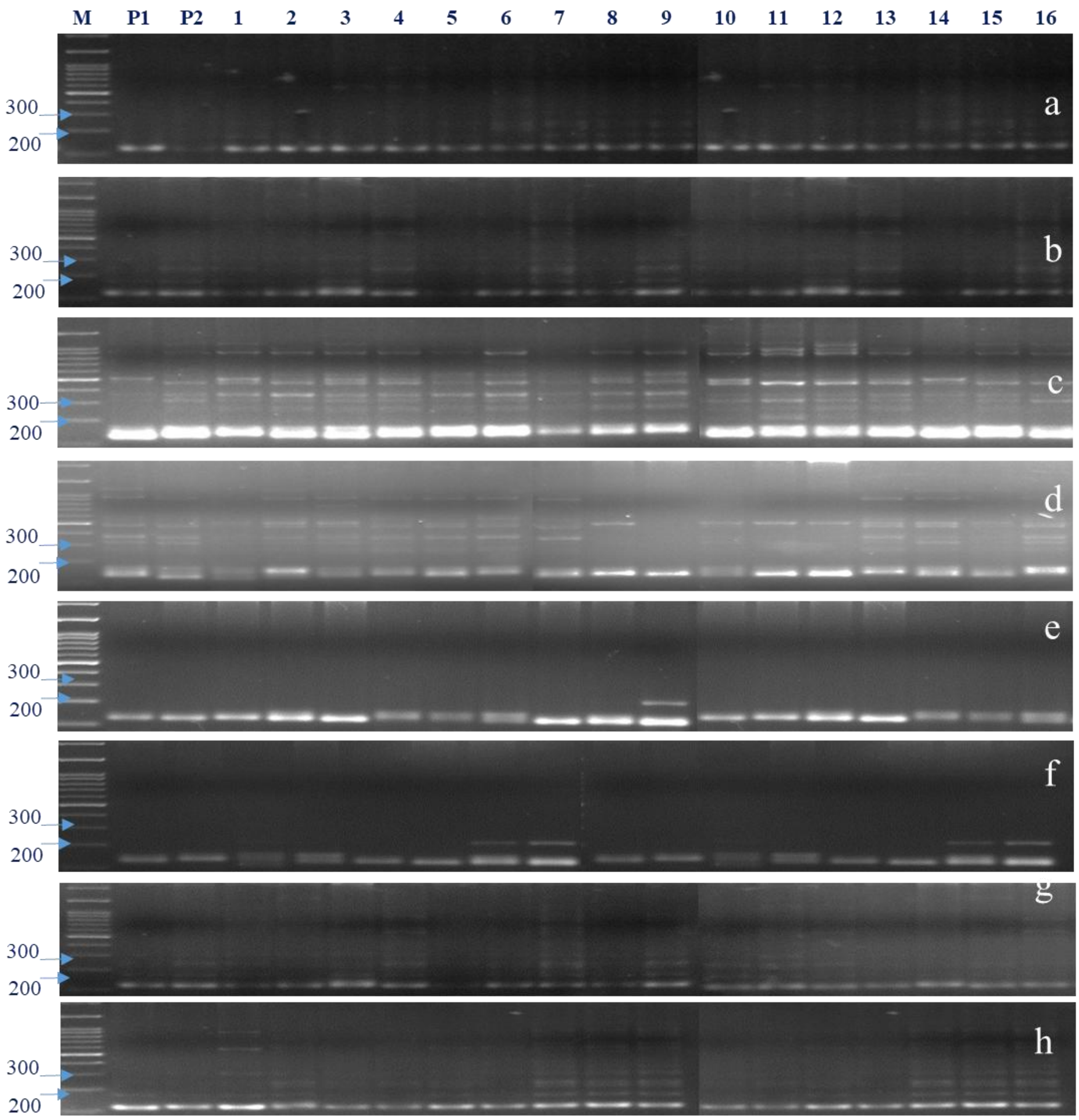

Supplementary figure 5m: Back-ground genome recovery analysis through in in BC_3F_3 generation (a) RM27451, (b) RM27840, (c) RM28130, (d) RM28270, (e) RM28767, (f) RM27789, (g) RM519, (h) RM28766

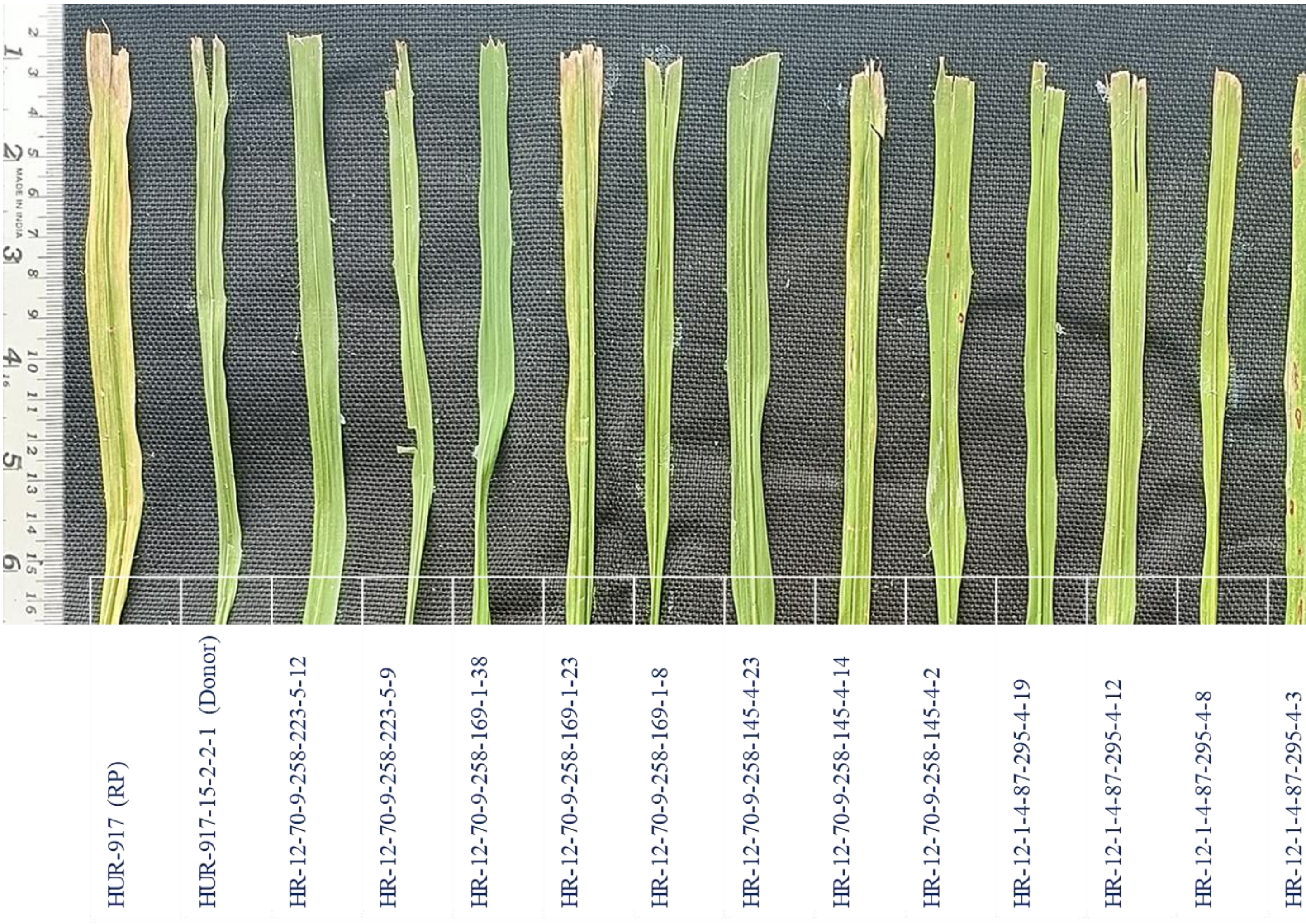

Supplementary Figure 6. Bacterial blight disease severity in parents and NILs after 21 days of inoculation of eight virulent *Xoo* races.
